# High-Power Impulse Magnetron Sputter Deposition of Ag on Self-Assembled Au Nanoparticle Arrays at Low-Temperature Dewetting Conditions

**DOI:** 10.1021/acsami.4c10726

**Published:** 2024-07-16

**Authors:** Tianfu Guan, Suzhe Liang, Yicui Kang, Evangelina Pensa, Dong Li, Wenkai Liang, Zhiqiang Liang, Yusuf Bulut, Kristian A. Reck, Tianxiao Xiao, Renjun Guo, Jonas Drewes, Thomas Strunskus, Matthias Schwartzkopf, Franz Faupel, Stephan V. Roth, Emiliano Cortés, Lin Jiang, Peter Müller-Buschbaum

**Affiliations:** † TUM School of Natural Sciences, Department of Physics, Chair for Functional Materials, Technical University of Munich, James-Franck-Str. 1, 85748 Garching, Germany; ‡ Nanoinstitute Munich, Faculty of Physics, Ludwig-Maximilians-Universität München, 80539 München, Germany; § Jiangsu Key Laboratory for Carbon-Based Functional Materials & Devices, Institute of Functional Nano & Soft Materials (FUNSOM), Soochow University, Suzhou 215123, P. R. China; ∥ Deutsches Elektronen-Synchrotron DESY, Notkestr. 85, 22607 Hamburg, Germany; ⊥ Chair for Multicomponent Materials, Department of Materials Science, Kiel University, 24143 Kiel, Germany; # Department of Fibre and Polymer Technology, KTH Royal Institute of Technology, Teknikringen 56-58, SE-100 44 Stockholm, Sweden

**Keywords:** plasmonic, sputter deposition, bimetallic nanostructure, *in situ*, grazing-incidence X-ray scattering

## Abstract

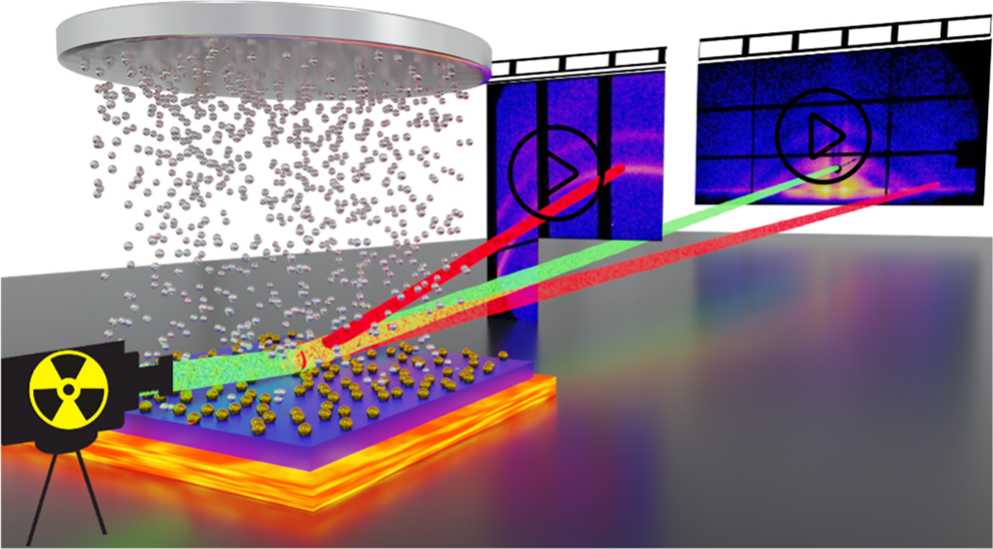

Plasmons have facilitated diverse analytical applications due to the boosting signal detectability by hot spots. In practical applications, it is crucial to fabricate straightforward, large-scale, and reproducible plasmonic substrates. Dewetting treatment, *via* applying direct thermal annealing of metal films, has been used as a straightforward method in the fabrication of such plasmonic nanostructures. However, tailoring the evolution of the dewetting process of metal films poses considerable experimental complexities, mainly due to nanoscale structure formation. Here, we use grazing-incidence small- and wide-angle X-ray scattering for the *in situ* investigation of the high-power impulse magnetron sputter deposition of Ag on self-assembled Au nanoparticle arrays at low-temperature dewetting conditions. This approach allows us to examine both the direct formation of binary Au/Ag nanostructure and the consequential impact of the dewetting process on the spatial arrangement of the bimetallic nanoparticles. It is observed that the dewetting at 100 °C is sufficient to favor the establishment of a homogenized structural configuration of bimetallic nanostructures, which is beneficial for localized surface plasmon resonances (LSPRs). The fabricated metal nanostructures show potential application for the surface-enhanced Raman scattering (SERS) detection of rhodamine 6G molecules. As SERS platform, bimetallic nanostructures formed with dewetting conditions turn out to be superior to those without dewetting conditions. The method in this work is envisioned as a facile strategy for the fabrication of plasmonic nanostructures.

## Introduction

Strongly enhanced light-matter interactions induced by localized surface plasmon resonance (LSPR) originate from electromagnetic fields excited in the junctions between metallic nanostructures.^1−3^ A strategic structure design can tune the plasmonic properties since the organization of metallic nanostructures offers the capacity to modulate plasmonic coupling interactions and thereby adjust their ability to enrich constructive hot spots.^4−6^ Due to these unique properties, metallic nanostructures are used for various applications such as solar conversion, analytics, data storage, and photocatalysis.^7−11^ It is widely recognized that LSPR characteristics largely depend on the metal type and interparticle distance within the nanogaps formed by the metallic nanostructure.^12−14^ To improve the plasmonic properties of these metallic nanostructures, an appropriate metallic composition and ensuring an optimal structural configuration are essential for enriching their applications. Among the metals utilized in these applications, gold (Au) and silver (Ag) received significant interest due to their exceptional plasmonic properties.^15,16^ Both Au and Ag exhibit minimal optical losses in the visible and near-infrared spectra, high polarizability, biocompatibility, and versatility.^17,18^ Specifically, Ag is known for its superior enhancement properties, while Au is known for its excellent stability.^19−21^ Another approach to enhancing the plasmonic properties involves integrating distinct metals onto a common substrate to construct a bimetallic or polymetallic plasmonic architecture.^22−24^ Unlike monometallic systems, bimetallic Au/Ag configurations combine the advantages of each metal, thereby improving their plasmonic properties.^25^ Furthermore, creating periodic nanostructures is crucial for achieving consistent plasmonic properties, which enhances reliability and expands their capability for a wide array of applications.^26,27^ Thus, it is essential to pursue more efficient and scalable fabrication techniques for plasmonic metallic nanostructures.

A dewetting treatment involving the thermal annealing of a metallic film to produce plasmonic nanostructures has found extensive use across various research contexts.^28,29^ Notably, this approach demonstrates its capability to efficiently manufacture metallic nanostructures on large surface areas. Furthermore, an inherent benefit of this method lies in its capacity to facilitate the creation of bimetallic structures by annealing distinct metallic films together, thereby yielding multifunctional materials. In recent years, many efforts have focused on the dewetting treatment to realize high-order plasmonic metal nanostructures.^30,31^ To name a few examples, Zheng et al.^28^ developed Au islands on the surface of fiber using a repeated dewetting technique at 600 °C, while Awasthi et al.^32^ fabricated Au nanoislands on polished silicon (Si) wafers using a similar thermal annealing process at 500 °C. In the realm of binary metallic structures, Kozioł et al.^33^ created Au/Ag nanoalloys through the thermal annealing of metallic films at 550 °C, and Li et al.^34^ proposed Ni/Au nanoparticles by rapidly thermal annealing Ni/Au bilayer heterofilms on a GaN layer at 550 °C. In general, the dewetting treatment can take place at temperatures well below the melting point, efficiently generating metallic nanostructures.^35,36^ Utilizing lower-temperature fabrication processes requires less energy input and thereby reduces fabrication costs in applications, offers improved control over experimental conditions, simplifies experimental setups, and reduces experimental waste. Thus, the quest for a low thermal annealing temperature remains an unresolved challenge in achieving a large-scale plasmonic platform with a highly organized structure and stable properties. This is essential as a prerequisite for technology adoption and practical applications. However, due to the stochastic nature of nucleation and growth processes inherent to the dewetting treatment without coupling to spinodal processes, precise control of the morphology of the metallic nanostructures remains challenging, thereby impeding the attainment of periodic configurations. The self-assembly method presents a comparably straightforward approach to fabricating periodic nanostructures.^37^ Moreover, depositing dissimilar metal films onto the self-assembled metal nanoarray enables the creation of multimetallic structures.

To fully maximize the functionality and application potential of the metal nanostructures, the monitoring of their formation process is essential. Hence, an *in situ* technique characterized by high-resolution capabilities to monitor the nanoscale evolutionary process is urgently necessary. Grazing-incidence X-ray scattering (GIXS) techniques are powerful methods for examining structures on the nanoscale.^38,39^ Due to their high-temporal-resolution data acquisition capacity and excellent sampling statistics, GIXS methods emerged as a widely favored approach for *in situ* experiments.^40,41^ In addition, GIXS methods can be used to investigate the dimensions, spatial distribution, and crystalline characteristics of metallic nanostructures during codeposition and growth.^42^ Thus, GIXS methods can be applied to monitor the metal film deposition process onto solid supports and thereby gain a fundamental understanding of the evolution of the formed metal nanostructures. Particularly, using the GIXS technique to examine commonly used metals in plasmonic structures, such as Au and Ag, is essential. Understanding the mechanisms of formation, evolution, and growth of these metal architectures by this technique is fundamental for exploring their potential as surface-enhanced Raman scattering (SERS) substrates and other practical applications.

To gain an in-depth study of such architectures, in the present study, we prepare bimetallic nanostructures on solid supports *via* high-power impulse magnetron sputter (HiPIMS) deposition of Ag onto templates consisting of Au nanoparticle (NP) arrays. During sputter deposition, dewetting conditions are realized by moderate heating of the substrate. With *in situ* monitoring of the sputter deposition *via* grazing-incidence small- and wide-angle X-ray scattering (GISAXS/GIWAXS), the growth characteristics are detailed. In particular, we compare templates with 10 nm (particle diameter) and 20 nm Au NP arrays under conditions with/without 100 °C thermal annealing. The morphology and crystalline characteristics of the bimetallic nanostructures are determined from the analysis of the GISAXS/GIWAXS data. The outcome of our study demonstrates that the Au/Ag composite exhibits an increased structural organization and enhanced crystalline properties when subjected to dewetting conditions. In addition, we can accurately trace the development of Au/Ag nanostructures by examining their size and distribution through our analytical GISAXS/GIWAXS approach. SERS measurements serve as a proof of concept for potential applications of such Au/Ag nanostructures, while the focus is not on the sensor optimization.

In the context of utilizing the Au/Ag nanocomposites with appropriately sized gaps for SERS applications, we opt for a deposition stage with a 2.5 nm gap size involving HiPIMS deposition of Ag for 10 s (effective thickness of *ca.* 3.06 nm). The SERS outcomes reveal that the 20 nm Au template, with dewetting conditions, exhibits the highest sensitivity in detecting rhodamine 6G (R6G) molecules. The SERS intensity at the 1365 cm^–1^ peak exhibits a 49% enhancement compared with the substrate without dewetting treatment. Thereby, this study provides a promising approach for producing binary or multicomponent metal nanostructures while maintaining precise traces of their dimensions and interparticle spacings. Accordingly, our discoveries can be a pioneering advancement in creating SERS platforms, color display technologies, catalysts, and other applications rooted in plasmonics. This technique and analysis method potentially find extensive applications in the realm of plasmonics and contribute to the diversification of analytical techniques in this field.

## Results and Discussion

**Figure 1:**
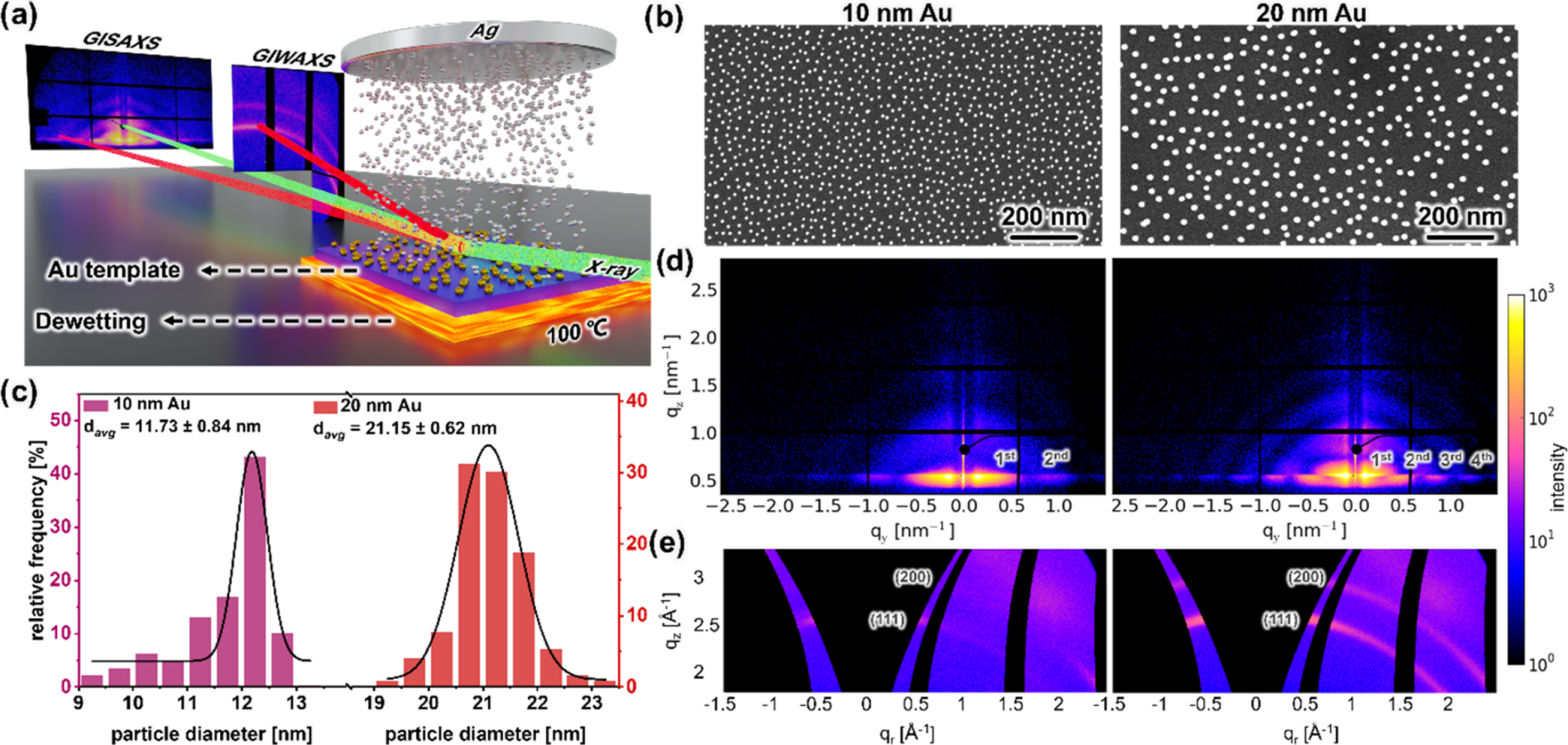
(a) Schematic of *in situ* HiPIMS deposition combined with in situ GIWAXS/GISAXS measurements. (b) SEM images of self-assembled 10 and 20 nm Au NPs arrays. (c) Particle diameter distributions of the 10 and 20 nm NP arrays extracted from SEM images by ImageJ. 2D (d) GISAXS and (e) GIWAXS data of both 10 and 20 nm Au NP arrays.

Tailoring the spatial arrangement of bimetallic nanostructures requires a robust approach to acquiring a profound comprehension of the nanostructure morphology. In addition, the study of the crystallinity of a bimetallic nanostructure is essential, as it impacts the stability and characteristics of the system.^43^ Consequently, the *in situ* investigation of morphology and crystallization becomes imperative for obtaining a nuanced understanding of the nanocomposites. Using the grazing-incidence geometry ensures real-time measurements with a high time resolution.^44,45^ Furthermore, this powerful technique, in combination with modeling, allows an in-depth understanding of the plasmonic nanocomposites.^46^Figures 1a and S1 show both the schematic and real configuration of the *in situ* measurement setup used in this study, respectively, which facilitates the investigation of both the crystalline structure (with GIWAXS) and the morphology on the nanoscale (with GISAXS) of the formed bimetallic nanostructures.

To study the influence of the templates on the nanocomposites, we assemble both 10 and 20 nm Au NP arrays on Si substrates. We examine the initial Au NP arrays before analyzing the Au/Ag binary structures induced *via* HiPIMS. Scanning electron microscopy (SEM) images (Figure 1b) reveal the well-organized assembly of Au NP arrays without any visible aggregations. Calculations of particle diameter and interparticle distance (as shown in Figures 1c and S2b) for the two template types resulted in average size values of (11.73 ± 0.84) and (21.15 ± 0.62) nm, with respective interparticle distances of (36.9 ± 2.9) and (60.7 ± 4.7) nm. For the sake of simplicity, we will continue to denote these templates *via* their particle sizes as 10 and 20 nm in the later context. The corresponding GISAXS data (Figure 1d) exhibits periodic Bragg scattering signals in horizontal scattering direction at the Yoneda peak position. In alignment with the SEM results, the observed scattering features indicate the remarkable size consistency and superior spatial distribution of the fabricated Au NP arrays. Furthermore, GIWAXS data (Figure 1e) shows the crystalline nature of the Au NPs, with distinct intensity rings located at *q*_*z*_ positions (*ca.* 2.64 and 3.05 Å^–1^) corresponding to the crystallographic planes (111) and (200) of Au.^47^ Notably, for the 10 nm Au NP arrays, a weak peak near the (111) plane suggests a suboptimal crystallization of gold. Conversely, the 20 nm Au NP array exhibits a pronounced crystalline quality, indicating its superior crystallization. Accordingly, we substantiate that the Au NP arrays produced through the self-assembly method show a high degree of organization, rendering them ideal templates for creating periodic bimetallic nanostructures.

**Figure 2:**
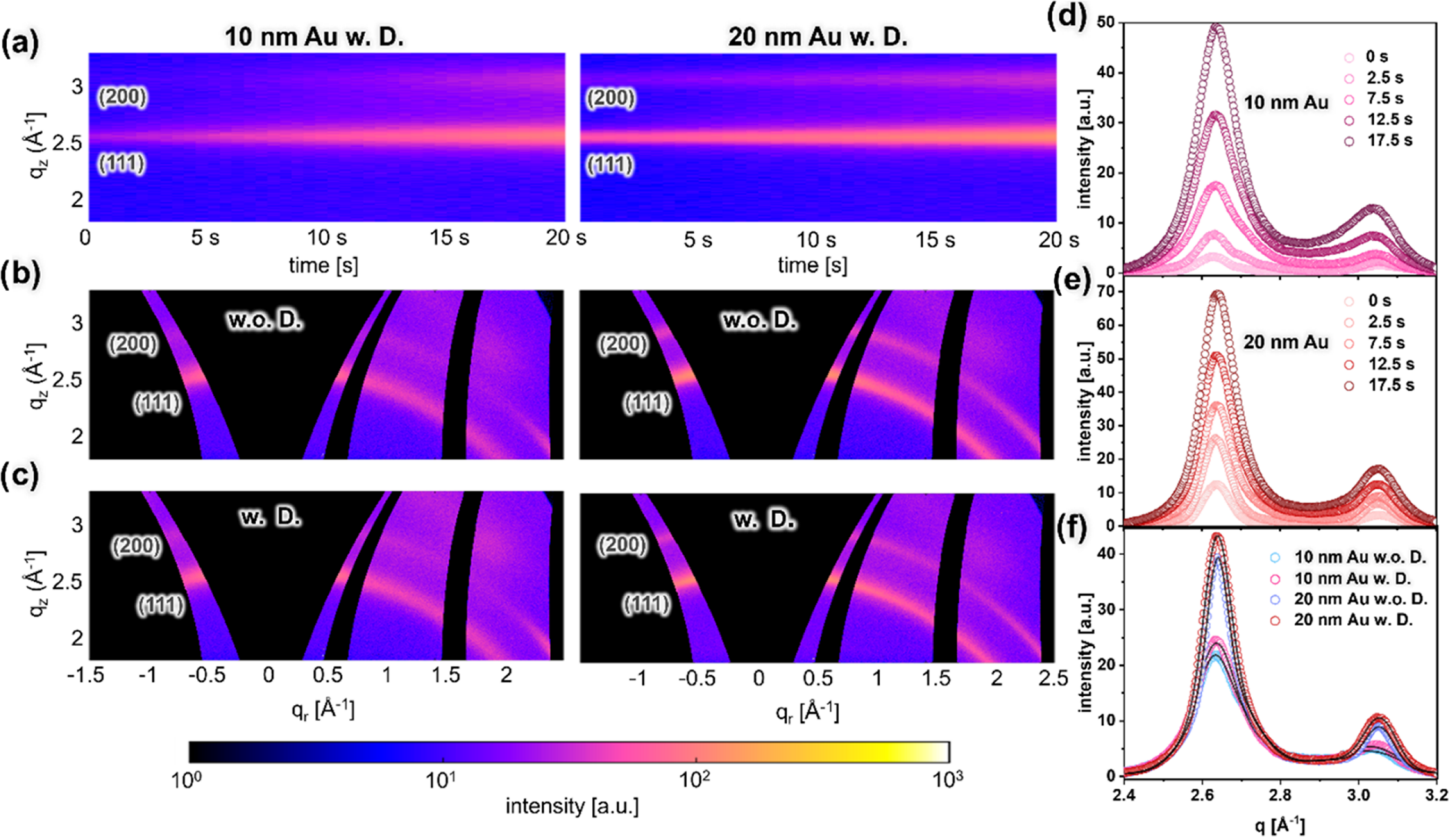
(a) Mappings of cake-cuts of the 2D GIWAXS data of 10 and 20 nm NP arrays acting as templates for the HiPIMS deposition of Ag under dewetting conditions. 2D GIWAXS data of (b) 10 nm and (c) 20 nm Au NP arrays after sputter deposition of Ag for 10 s (*ca.* 3.06 nm) without (w.o.) and with (w.) dewetting conditions. (d) Azimuthal integrations of selected 2D GIWAXS data collected during the sputter deposition of Ag on (d) 10 nm and (e) 20 nm NP arrays with dewetting treatment. (f) Azimuthal integrations of the 2D GIWAXS data shown in (b, c); the black line is the corresponding fit for both (111) and (200) peaks.

Aside from Au, Ag is another well-liked noble metal renowned for its beneficial plasmonic characteristics.^48^ Consequently, we choose Ag as a second metallic constituent in creating Au/Ag nanostructures *via* the HiPIMS deposition method on the Au NP arrays (deposition rate *ca.* 3.06 Å/s). To attain improved uniformity within the binary plasmonic architecture, we perform an *in situ* thermal annealing process at the low temperature of 100 °C during the sputter deposition. Utilizing these thermal dewetting conditions has already found application in various research for creating plasmonic nanostructures.^49^ Since the thermal annealing is applied during the sputter deposition in the present study, only a more moderate temperature is required. Actually, the annealing temperature applied in the present study is lower than the temperatures in earlier reports (Table S1). Figures 2a and S3 illustrate the temporal evolution of GIWAXS data for 10 and 20 nm template samples with the HiPIMS deposition. Similar to the GIWAXS results of the bare Au NPs, two distinct intensity peaks in the GIWAXS data correspond to the (111) and (200) crystallographic planes of Au/Ag. As the duration of Ag deposition increases, there is a consistent augmentation in the intensity of these peaks. Notably, the observed constancy in peak positions can be attributed to the Ag crystal planes close to Au, resulting in the absence of significant shifts in peak positions. Figures 2b,c and S4 show selected two-dimensional (2D) GIWAXS data of both 10 and 20 nm templates, with different HiPIMS deposition times of Ag.

To analyze the intensity evolution of crystallographic planes during sputtering, we make cake-cuts (Figure S4) of the 2D GIWAXS data. The respective one-dimensional (1D) data are seen in Figures 2d,e and S5. Two distinct Bragg peaks indicate the (111) and (200) planes of Au/Ag. Consistent with previous reports, the intensity of the (111) peak is stronger than that of the (200) peak. In the case of the 10 nm template sample, the weak peak close to the (111) position gradually disappears with increasing sputter time, indicating that the crystallization of the bimetallic nanostructure is enhanced. Significantly, during the HiPIMS deposition under dewetting conditions, the two Bragg peaks show a higher intensity in contrast to the sample without dewetting conditions (Figure 2f). This difference shows that thermal annealing affects the crystallization of the bimetallic nanostructures. Notably, the crystallization of the metals impacts their stability and plasmonic characteristics. The validation of this phenomenon contributes to the confidence in using low-temperature thermal annealing conditions as a viable technique for fabricating plasmonic nanostructures.

**Figure 3:**
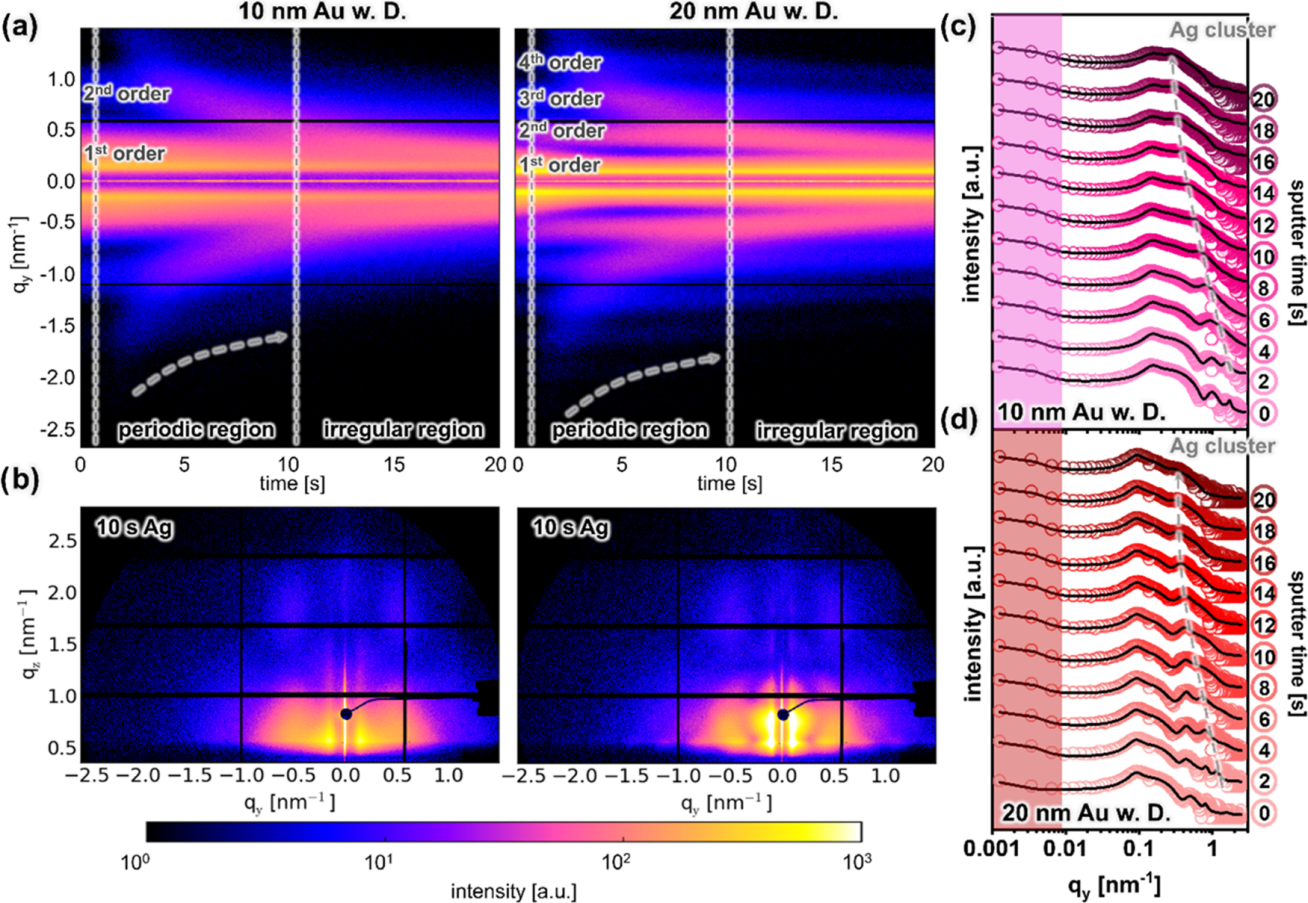
(a) Mappings of horizontal line cuts from 2D GISAXS data of 10 and 20 nm templates during the HiPIMS deposition of Ag under dewetting conditions. (b) 2D GISAXS data of 10 and 20 nm Au templates with Ag sputter deposited for 10 s (effective thickness of *ca.* 3.06 nm) with dewetting conditions. Selected horizontal line cuts from 2D GISAXS data (symbols) are shown together with fits (lines) of both (c) 10 nm and (d) 20 nm Au templates during the *in situ* sputter deposition of Ag under dewetting conditions. The position of the line cuts is indicated in Figure S6.

In addition to the crystallization characteristics, precise spatial control is another crucial aspect of outstanding plasmonic nanostructures. This aspect is particularly important in the plasmonic structure fabrication, where an in-depth analysis of the dimensions and interparticle gaps within the metallic structure directly enables the creation of an optimal configuration for hot spots.^50,51^ Hence, it is imperative to systematically examine the formation of binary metallic nanostructures by using high-resolution techniques in conjunction with modeling methodologies aiming at the accurate reconstruction of the nanostructure. Utilizing the GISAXS geometry is a pivotal approach to realize this objective.^38^ A selection of *in situ* GISAXS data acquired under varying HiPIMS deposition durations is shown in Figure S6 and Video 1. The statistical analysis of the Au/Ag bimetallic nanostructure formation process is carried out by reducing the 2D GISAXS data into 1D representations *via* horizontal line cuts. Figures 3a and S7 exhibit time-dependent mappings composed of horizontal line cuts at the Yoneda peak position of Au/Ag (Figure S6). We delineate the temporal evolution of critical scattering features, thereby revealing two overarching structural states, which are periodic and irregular regions.

Right from the start of the sputter deposition process, in the mappings equidistant peaks are seen across all examined samples. In particular, there are two obvious peaks for 10 nm and four peaks for 20 nm Au NP arrays. These peaks emanate from the organized array of Au NPs and arise from the mean interparticle distance within the Au nanostructure. As the sputter deposition progresses, an additional Bragg peak feature (highlighted by the dashed line) emerges, which is primarily attributed to the presence of Ag clusters. This Ag cluster peak moves toward smaller *q*_*y*_ values throughout the sputter deposition, signifying an increase in the mean interparticle distance and particle size. Additionally, the first-order peak associated with the Au NPs tends to become more narrow, suggesting that the deposited Ag increases the size of the Au/Ag composite nanoparticles. After about 10 s sputter deposition of Ag (effective thickness of *ca.* 3.06 nm), the second-order peak of the 10 nm template merged into the first-order peak.

A comparable phenomenon is observable in the case of the 20 nm sample, where the third-order peak gradually merges with the second-order peak during the Ag deposition process. This outcome implies that following the deposition of Ag for approximately 10 s, the bimetallic nanostructure exhibits a notably reduced order in its structural arrangement. Consequently, in the present study, 10 s HiPIMS deposition is identified as the boundary demarcating the periodic region of the Au/Ag bimetallic nanostructures. The 2D GISAXS data (Figure 3b, 10 and 20 nm template with deposition Ag for 10 s under dewetting conditions) substantiates this observation by the absence of periodic Bragg features when compared to the results obtained with the bare Au NP arrays (Figure 1d). Notably, the 20 nm Au template displays more periodic intensity peaks in GISAXS in comparison to the 10 nm template, suggesting a higher level of structure order when depositing Ag for 10 s. This outcome can be ascribed to the template’s larger size, which experiences a relatively minor influence from the Ag deposition, allowing it to maintain a more periodic structure. In the context of plasmonic nanostructure application, achieving a high order of the nanostructure is significant, as it contributes to generating periodic hot spots, thereby enhancing the plasmonic properties.^52^ Furthermore, achieving reduced interparticle spacings is also imperative for creation of hot spots. Hence, the choice of an appropriate nanostructure holds utmost significance. In this particular instance, we designate the 10 s deposition as the preferred stage due to its capacity to yield the narrowest interstitial spacing within the periodic region of our HiPIMS deposition process.

To quantitatively assess the spatial characteristics of the Au/Ag bimetallic nanostructures in a precise manner, we analyze the GISAXS data with a theoretical framework based on the distorted wave Born approximation (DWBA) (Figure S8) for the accurate determination of particle dimensions and interparticle gaps. Figures 3c,d and S9 depict the selected horizontal line cuts of the 2D GISAXS data, taken every 2 s (an interval of effective thickness of *ca.* 0.61 nm). Experimental data (symbols) are compared with the corresponding model fits (black lines) for both the 10 and 20 nm templates subjected to Ag deposition. The pure Au NP arrays (bottom curves), characterized by clearly visible scattering peaks, exhibit a well-defined periodic structure. Within a short period after initiating the sputtering process (as shown in the curve at 2 s), a prominent peak emerges at a high *q*_*y*_ value (*ca.* 1.6 nm^–1^), signifying the scattering signature of the Ag nanostructure. As the sputter deposition continues, the scattering peak associated with Ag progressively shifts toward lower *q*_*y*_ values, indicating the growth of Ag clusters *via* coalescence (gray arrow). Furthermore, the regularly spaced peaks resulting from the Au NP array become less distinct, by transitioning from a sharp peak to a more diffuse shoulder-like scattering feature after 10 s of sputter deposition. This change suggests that the binary structure undergoes a loss of well-defined periodicity when subjected to longer deposition times of Ag in HiPIMS. It is worth noting that the intensity of the peaks originating from the Ag clusters on both 10 and 20 nm Au templates is significantly enhanced under dewetting conditions (Figure S10). The intensities in the GISAXS data predominantly signify the number of scatterers and their order. In our context, these increased intensities result from a higher degree of structural order, which will be beneficial for the SERS properties.

**Figure 4:**
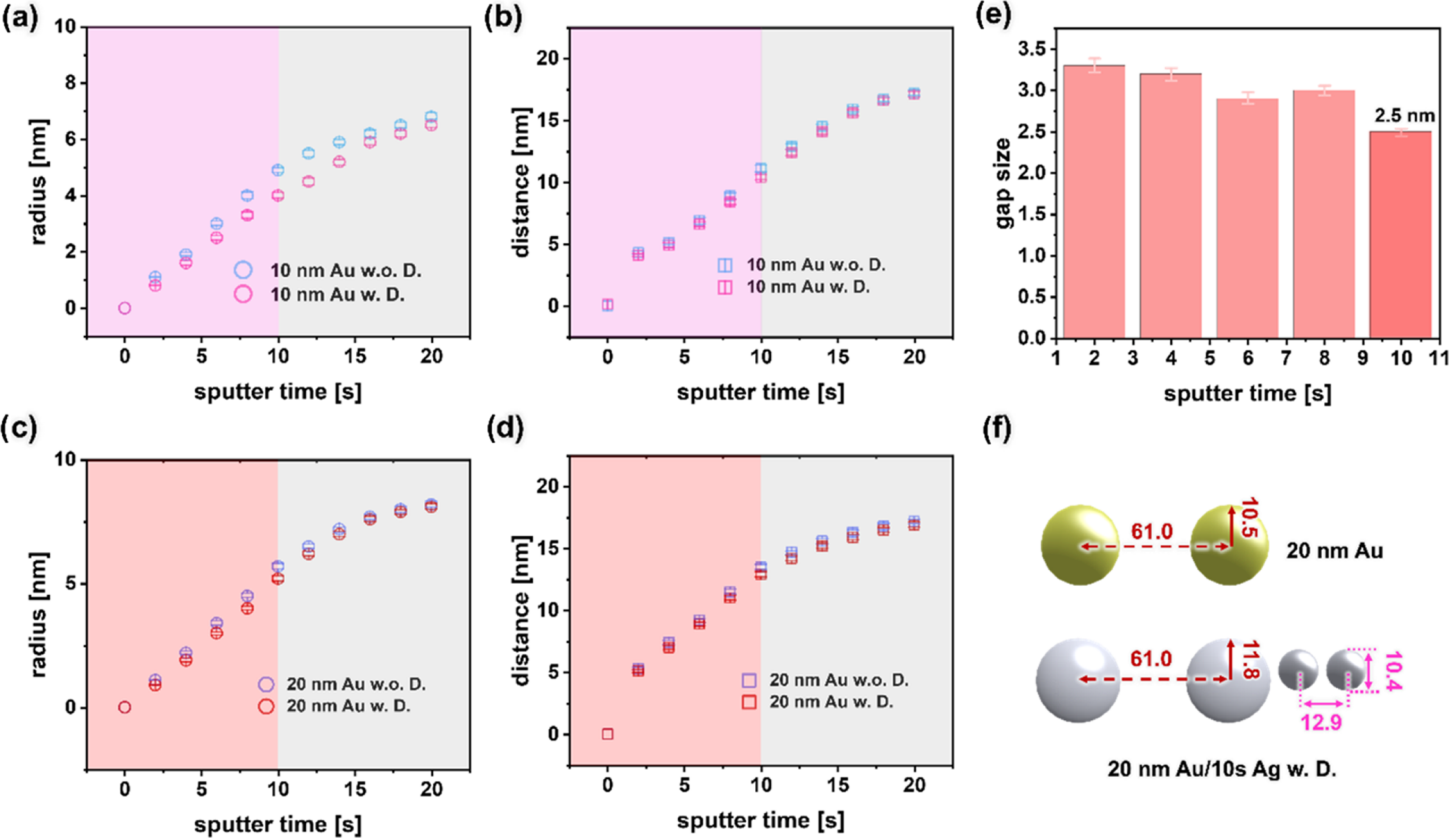
(a, c) Radius and (b, d) interparticle distance of Ag NPs located between the Au NPs acting as template during the sputter deposition in case of the (a, b) 10 and (c, d) 20 nm templates as extracted from GISAXS fits. (e) Gap sizes between Ag NPs of 20 nm template calculated from determined parameters. (f) Schematic structures (top view) of bare 20 nm Au NPs before (top) and after sputter deposition of Ag for 10 s Ag (bottom) as determined from the fit parameters.

The evolution of fit parameters, encompassing the sizes and interparticle distances of the Au/Ag bimetallic nanostructures, is seen in Figures 4 and S11. These modeling results are consistent with the radius and interparticle distance distribution analysis of the SEM images (Figures 1c and S2b), which validates the reliability of our GISAXS modeling. Figure S11a,b demonstrate that the interparticle spacing remains consistent for both the 10 and 20 nm Au templates, throughout the Ag deposition process. This consistency suggests that the Au NPs serve as periodically positioned nucleation centers for the bimetallic nanostructure formation. Additionally, an increase in the size of the Au/Ag structure as the sputtering time is extended affirms the successful formation of the binary structure (Figure S11c,d). The sizes of the Au/Ag nanostructures achieved under the dewetting conditions are smaller compared to those without dewetting conditions. This finding suggests that the thermal annealing of the substrate contributes to a more regular arrangement of the formed NPs. Figure 4a–d provides information on the radius and center-to-center distance of bare Ag NPs, which are isolated between the larger Au/Ag NPs. The spacing between these Ag NPs on both the 10 and 20 nm Au templates increases as the sputter time progresses. Notably, the Ag NPs subjected to dewetting conditions exhibit a reduced radius and interparticle distance in comparison to those without dewetting conditions. These outcomes can be ascribed to the effects of thermally activated surface diffusion of Ag NPs. In the course of the sputter deposition, the formation of Ag structures initiates from individual island grains, rather than originating from a continuous film.^53^ Motivated to minimize the surface energy, the Ag islands subjected to dewetting exhibit a more homogeneous formation process, gradually transforming into regular NPs. Consequently, these regular Ag NPs show smaller dimensions than their irregular counterparts and feature a higher density of nucleation sites.

For plasmonic applications, the gaps within the plasmonic structures play a crucial role in generating hot spots. Figure 4e presents the computed gaps determined from the modeling parameters. Our study can achieve exceptionally narrow gaps (*ca.* 2.5 nm). In contrast to prior investigations, in a SERS platform, such a sub-3 nm gap, as realized within our study, demonstrates a notable competitiveness.^33^ Additionally, a top-view schematic of the model used for the GISAXS modeling (Figure 4f) elucidates both the interparticle spacing and dimensions of bare 20 nm Au NP array and their configuration following a 10 s HiPIMS deposition of Ag.

**Figure 5:**
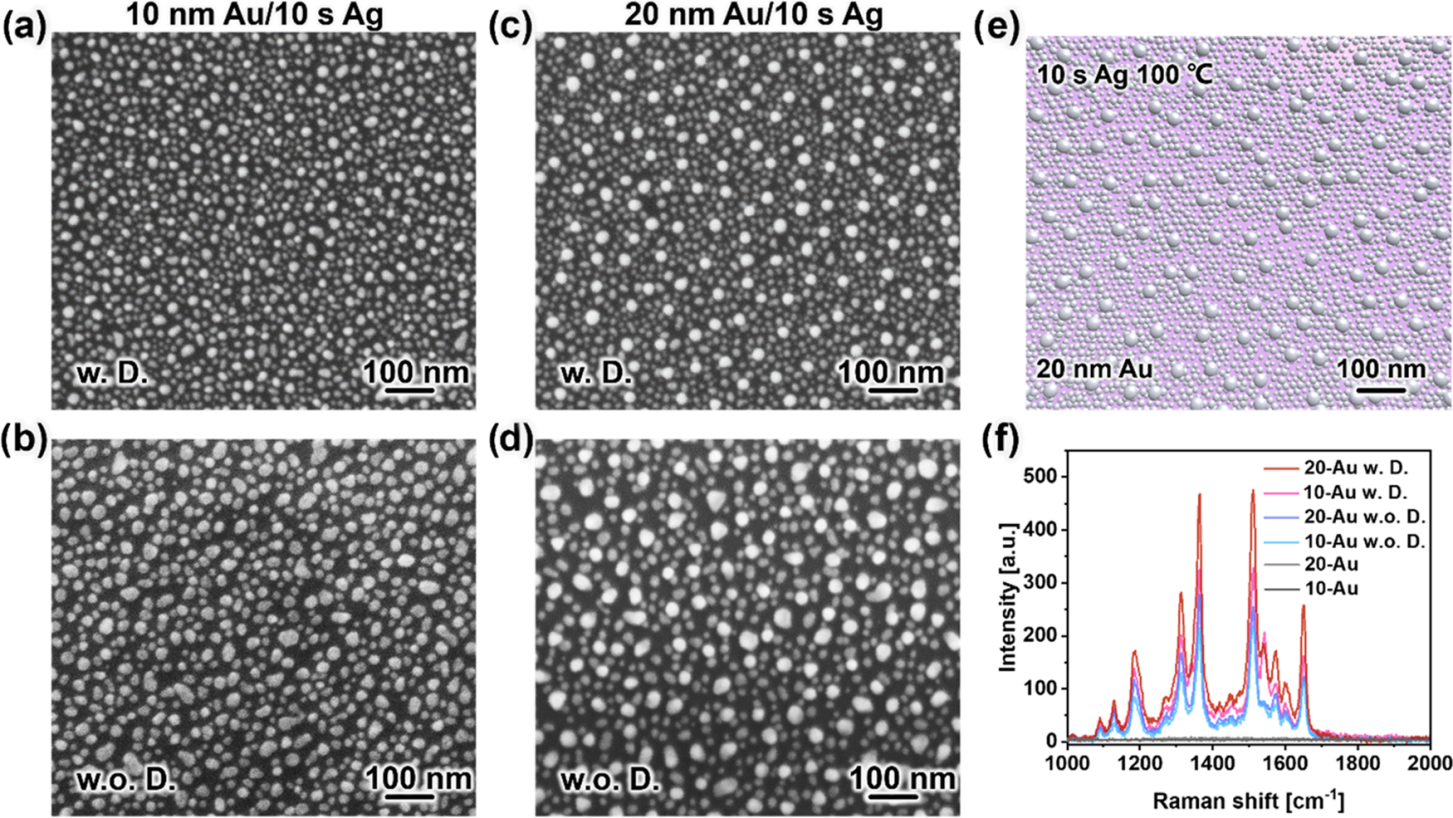
SEM images of (a, b) 10 nm and (c, d) 20 nm Au templates after HiPIMS deposition of Ag for 10 s (a, c) with and (b, d) without dewetting conditions. (e) Schematics of 20 nm Au template sputtered with 10 s Ag under dewetting conditions. (f) SERS results of 10^–4^ M R6G molecule detected by 10 and 20 nm Au templates sputtered 10 s Ag with and without dewetting conditions.

To further corroborate these results, we conduct *ex situ* SEM measurements on the Au template with Ag deposition for 5, 10, and 20 s. These SEM images reveal discernible differences due to the applied dewetting conditions, suggesting that our choice of a low-temperature thermal treatment at 100 °C is sufficient to yield substantial outcomes (Figures 5a–d, S12, and S13). Figure 5a–d depicts both 10 and 20 nm Au templates with Ag deposition for 10 s. Consistent with prior findings, it is evident that the Au/Ag bimetallic nanostructure exhibits a more ordered arrangement under dewetting conditions than the sample that did not undergo the heating process. In addition, it is notable that the number density of the Ag NPs located between the large Au/Ag NPs is larger when using dewetting conditions. Significantly, the 20 nm template exhibits an improvement of regularity of its structure when contrasted with the 10 nm template, rendering it a more desirable choice for potential applications in SERS. Furthermore, we reversely model the geometry 20 nm Au template with a 10 s Ag deposition bimetallic nanostructure through the utilization of modeling parameters (Figure 5e). The results of the modeling align with the observations from SEM, further validating our GISAXS modeling approach.

To substantiate the potential use of the bimetallic nanostructures analyzed in this study, we conduct SERS measurements on the samples of Au template with HiPIMS deposition of Ag for 10 s. Rhodamine 6G (R6G) is commonly used in SERS applications, with the detection method leveraging the technique’s notable sensitivity and selectivity, offering rapid and nondestructive on-site analysis.^54−57^ In this work, we choose R6G as the model molecule to be detected with SERS. The spectral signals from the molecules are distinctly discernible at a concentration of 10^–4^ M (Figure 5f). Notably, in comparison to the sample that was not subjected to the heating process, the SERS signal is increased. The 20 nm Au substrates with dewetting condition exhibit a SERS intensity at the 1365 cm^–1^ peak with a 49% enhancement compared with the substrate without dewetting treatment. This observation strongly implies that the dewetting conditions foster the formation of a more regular bimetallic nanostructure, consequently leading to a notable enhancement in the SERS properties. Additionally, we analyze the relative standard deviation (RSD) of 20 nm Au/10 s Ag substrates concerning the SERS peak intensities at 1365 and 1512 cm^–1^. The substrate with dewetting conditions yielded an RSD of 6.34 and 5.96%, respectively (Figure S14a), reflecting a good signal reproducibility when compared with the substrate without dewetting conditions (Figure S14b). It is worth mentioning that the primary objective of our research is to investigate the formation mechanisms of Au/Ag bimetallic nanostructures through our techniques rather than optimizing SERS. Consequently, the detection of practical molecules and the calculation of the limit of detection (LOD) were not explored in this study.

## Conclusions

In summary, this study outlines the fabrication of periodic binary metal nanostructures composed of Au and Ag through the HiPIMS deposition of Ag onto a self-assembled array of Au NPs under dewetting conditions. To gain insights into the formation mechanisms of the bimetallic nanostructures, we use *in situ* X-ray scattering techniques. We investigate the morphology of 10 and 20 nm Au templates following HiPIMS deposition of Ag with both GIWAXS and GISAXS. The GIWAXS results reveal an augmented crystallization of the binary Au/Ag architecture due to the dewetting conditions installed by the heated substrate. Through the subsequent modeling of GISAXS data, we derive information about the dimensions and center-to-center distance of nanostructures at various stages of the HiPIMS deposition process. Upon computation of the interparticle gaps through modeling, we attain an optimal configuration (Ag deposition for 10 s, effective thickness of *ca.* 3.06 nm) suitable as a SERS platform. By using these binary Au/Ag nanostructures to detect R6G, we observe that 20 nm Au Np array/10 s Ag in combination with the dewetting conditions yields the highest detectivity. These results elucidate a prospective approach for both the fabrication and analysis of binary plasmonic nanostructures, which is offering promise as an effective SERS platform. The systematic analysis of these binary structures furnishes significant insights for designing and assessing plasmonic nanostructures. Thereby, our findings provide a promising candidate for advancing binary plasmonic nanostructures, including SERS platforms, color display technologies, catalysts, and related domains.

## Experimental Section

### Materials

Chloroauric acid (HAuCl_4_, 99.9%), sodium borohydride (NaBH_4_), l-ascorbic acid (AA), cetyltrimethylammonium bromide (CTAB), cetyltrimethylammonium chloride (CTAC, 96%), 3-aminopropyltriethoxysilane (APTES), succinic anhydride, and Rhodamine 6G (R6G) were purchased from Sigma-Aldrich. Hydrogen peroxide (H_2_O_2_, 30%) and sulfuric acid (H_2_SO_4_, 98%) were purchased from Carl Roth GmbH & Co., KG. Silicon wafers (Si 100, p-type) were purchased from Silchem Handels GmbH (Freiberg, Germany). Silver (99.99%) was purchased from MaTeck GmbH (Jülich, Germany).

### Synthesis and Self-Assembly of Au NPs

Both 10 and 20 nm Au NPs were synthesized through the seed-mediated growth method. Au nanoseeds, around 3–5 nm, were gained by quickly injecting NaBH_4_ solution (0.01 M, 0.6 mL) into the mixed solution of CTAB (0.1 M, 9.75 mL) and HAuCl_4_ (0.01 M, 0.25 mL). By adding as-prepared nanoseeds (0.3 mL) into a mixed solution containing H_2_O (190 mL), CTAB (0.1 M, 9.75 mL), HAuCl_4_ (0.01 M, 4 mL), and AA (0.1 M, 15 mL), 10 nm Au NPs were obtained. 20 nm Au NPs were fabricated with the same process by changing the seed contents to 0.15 mL. After standing overnight, the solutions were centrifuged (15,000 rpm, 7 min for 10 nm; 10,000, 5 min for 20 nm) twice to rinse off extra surfactant and redispersed in deionized (DI) water for the next steps.

As for the surface modification of substrates, the Si wafers were cleaned by an acid bath (H_2_SO_4_/H_2_O_2_ = 7:3). After cleaning with deionized water, the substrates were immersed in 1% APTES/ethanol solution for 8 h to grafted amino groups. Then, the substrate was put into 0.01 M succinic anhydride/ethanol solution for 8 h to modify the negative charge. In the end, the self-assembled Au NSs monolayer arrays were acquired by immersing the modified substrates into both 10 and 20 nm Au colloidal solutions for about 6 h. To ensure that all chemical coatings were completely removed from the substrates and Au NPs after self-assembly, we washed the substrates several times with ethanol and annealed them at 100 °C for 10 min every time.

### *In Situ* Measurements

The *in situ* GIWAXS/GISAXS measurements were performed at the MiNaXS/P03 beamline of the PETRA III storage ring at DESY, Hamburg.^58^ The scattering signals were collected with a LAMBDA 9 M (2 images per second, pixel size 55 μm, X-Spectrum, Germany) and Pilatus 2 M (20 images per second, pixel size 172 μm, Dectris Ltd., Switzerland) detector, respectively. The detailed instrumental parameters of the GIWAXS/GISAXS setup can be found in Table S2. The sputter deposition (deposition rate 0.306 nm/s) measurements with a 100 °C heating process were performed by the HiPIMS system and followed *in situ* with GIXS measurements. The sample was moved repeatedly perpendicular to the X-ray beam along the horizontal direction during the *in situ* measurements to avoid possible X-ray radiation damage of the sample. The schematic and real configuration of the *in situ* measurements setup are shown in Figures 1a and S1.

### Characterization

The field emission SEM (FESEM) images were obtained by a high-resolution FESEM (Zeiss Gemini NVision 40, Germany) at a working distance of 5 mm and an accelerating voltage of 5 kV. For the SERS measurements, we performed *ex situ* HiPIMS deposition of Ag on the Au NP substrates. The bimetallic nanostructure-coated substrates were immersed in an R6G 10^–4^ M solution and left overnight at room temperature. Then, the substrates were removed from the solution, rinsed with ultrapure water, left to dry at room temperature, and placed under the microscope. SERS spectra were acquired with a Witec spectrometer (Germany), using a 100× air objective and the 633 nm wavelength under a power of 0.5 mW provided by a He–Ne laser, with an integration time of 2 s.

## Supporting Information Available

*In situ* GISAXS data (MP4)
Photograph of in situ measurement setup; GISAXS and GIWAXS data; GISAXS data modeling details; SEM image; RSD of SERS results; temperature comparison with previous work, and GIXS setup parameters (PDF)

## References

[ref1] Mayer K. M.; Hafner J. H. Localized Surface Plasmon Resonance Sensors. Chem. Rev. 2011, 111, 3828–3857. 10.1021/cr100313v. 21648956

[ref2] Azzam S. I.; Kildishev A. V.; Ma R. M.; Ning C. Z.; Oulton R.; Shalaev V. M.; Stockman M. I.; Xu J. L.; Zhang X. Ten Years of Spasers and Plasmonic Nanolasers. Light: Sci. Appl. 2020, 9, 90 10.1038/s41377-020-0319-7. 32509297 PMC7248101

[ref3] Zhang J.; Wang Y.; Li D.; Sun Y.; Jiang L. Engineering Surface Plasmons in Metal/Nonmetal Structures for Highly Desirable Plasmonic Photodetectors. ACS Mater. Lett. 2022, 4 (2), 343–355. 10.1021/acsmaterialslett.1c00768.

[ref4] Boltasseva A.; Atwater H. A. Low-Loss Plasmonic Metamaterials. Science 2011, 331, 290–291. 10.1126/science.1198258. 21252335

[ref5] Ha M.; Kim J. H.; You M.; Li Q.; Fan C.; Nam J. M. Multicomponent Plasmonic Nanoparticles: From Heterostructured Nanoparticles to Colloidal Composite Nanostructures. Chem. Rev. 2019, 119, 12208–12278. 10.1021/acs.chemrev.9b00234. 31794202

[ref6] Guo C.; Yu J.; Deng S. Hybrid Metasurfaces of Plasmonic Lattices and 2D Materials. Adv. Funct. Mater. 2023, 33, 2302265 10.1002/adfm.202302265.

[ref7] Yang H.; Li D.; Zheng X.; Zuo J.; Zhao B.; Li D.; Zhang J.; Liang Z.; Jin J.; Ju S.; Peng M.; Sun Y.; Jiang L. High Freshwater Flux Solar Desalination via a 3D Plasmonic Evaporator with an Efficient Heat-Mass Evaporation Interface. Adv. Mater. 2023, 35, 2304699 10.1002/adma.202304699. 37524107

[ref8] Itoh T.; Prochazka M.; Dong Z. C.; Ji W.; Yamamoto Y. S.; Zhang Y.; Ozaki Y. Toward a New Era of SERS and TERS at the Nanometer Scale: From Fundamentals to Innovative Applications. Chem. Rev. 2023, 123, 1552–1634. 10.1021/acs.chemrev.2c00316. 36745738 PMC9952515

[ref9] Yang J.; Zhang X.; Zhang X.; Wang L.; Feng W.; Li Q. Beyond the Visible: Bioinspired Infrared Adaptive Materials. Adv. Mater. 2021, 33 (15), 2004754 10.1002/adma.202004754. 33624900

[ref10] Gao Y.; Cheng F.; Fang W.; Liu X.; Wang S.; Nie W.; Chen R.; Ye S.; Zhu J.; An H.; Fan C.; Fan F.; Li C. Probing of Coupling Effect Induced Plasmonic Charge Accumulation for Water Oxidation. Nati. Sci. Rev. 2021, 8, nwaa151 10.1093/nsr/nwaa151. PMC8288172 34691655

[ref11] Stefancu A.; Gargiulo J.; Laufersky G.; Auguié B.; Chiş V.; Le Ru E. C.; Liu M.; Leopold N.; Cortés E. Interface-Dependent Selectivity in Plasmon-Driven Chemical Reactions. ACS Nano 2023, 17, 3119–3127. 10.1021/acsnano.2c12116. 36722817

[ref12] Lim D. K.; Jeon K. S.; Hwang J. H.; Kim H.; Kwon S.; Suh Y. D.; Nam J. M. Highly Uniform and Reproducible Surface-enhanced Raman Scattering from DNA-tailorable Nanoparticles with 1-nm Interior Gap. Nat. Nanotechnol. 2011, 6, 452–460. 10.1038/nnano.2011.79. 21623360

[ref13] Bell S. E. J.; Charron G.; Cortés E.; Kneipp J.; Chapelle M. L.; Langer J.; Procházka M.; Tran V.; Schlgcker S. Towards Reliable and Quantitative Surface-Enhanced Raman Scattering (SERS): From Key Parameters to Good Analytical Practice. Angew. Chem., Int. Ed. 2020, 59, 5454–5462. 10.1002/anie.201908154. PMC7154527 31588641

[ref14] Chen Y.; Zhu D.; Zhong H.; Gan Z.; Zong S.; Wang Z.; Cui Y.; Wang Y. Ultrasensitive Detection of Matrix Metalloproteinase 2 Activity Using a Ratiometric Surface-Enhanced Raman Scattering Nanosensorwith a Core-Satellite Structure. ACS Appl. Mater. Interfaces 2024, 16 (3), 4160–4168. 10.1021/acsami.3c15344. 38204415

[ref15] Peng J.; Lin Q.; Foldes T.; Jeong H. H.; Xiong Y.; Pitsalidis C.; Malliaras G. G.; Rosta E.; Baumberg J. J. In-Situ Spectro-Electrochemistry of Conductive Polymers Using Plasmonics to Reveal Doping Mechanisms. ACS Nano 2022, 16, 21120–21128. 10.1021/acsnano.2c09081. 36468680 PMC9798863

[ref16] Juang R.-S.; Wang K.-S.; Cheng Y.-W.; Fu C.-C.; Chen W.-T.; Liu C.-M.; Chien C.-C.; Jeng R.-J.; Chen C.-C.; Liu T.-Y. Floating SERS Substrates of Silver Nanoparticles-Graphene Based Nanosheets for Rapid Detection of Biomolecules and Clinical Uremic Toxins. Colloids Surf., A 2019, 576, 36–42. 10.1016/j.colsurfa.2019.05.042.

[ref17] Son W. K.; Choi Y. S.; Han Y. W.; Shin D. W.; Min K.; Shin J.; Lee M. J.; Son H.; Jeong D. H.; Kwak S. Y. In Vivo Surface-enhanced Raman Scattering Nanosensor for the Real-time Monitoring of Multiple Stress Signalling Molecules in Plants. Nat. Nanotechnol. 2023, 18, 205–216. 10.1038/s41565-022-01274-2. 36522556

[ref18] Chen Y. W.; Liu T. Y.; Chen P. J.; Chang P. H.; Chen S. Y. A High-Sensitivity and Low-Power Theranostic Nanosystem for Cell SERS Imaging and Selectively Photothermal Therapy Using Anti-EGFR-Conjugated Reduced Graphene Oxide/Mesoporous Silica/AuNPs Nanosheets. Small 2016, 12 (11), 1458–1468. 10.1002/smll.201502917. 26814978

[ref19] Zhu R.; Feng H.; Li Q.; Su L.; Fu Q.; Li J.; Song J.; Yang H. Asymmetric Core–Shell Gold Nanoparticles and Controllable Assemblies for SERS Ratiometric Detection of MicroRNA. Angew. Chem., Int. Ed. 2021, 60, 12560–12676. 10.1002/anie.202102893. 33769682

[ref20] Yang M.-C.; Hardiansyah A.; Cheng Y.-W.; Liao H.-L.; Wang K.-S.; Randy A.; Harito C.; Chen J.-S.; Jeng R.-J.; Liu T.-Y. Reduced Graphene Oxide Nanosheets Decorated with Core-Shell of Fe_3_O_4_-Au Nanoparticles for Rapid SERS Detection and Hyperthermia Treatment of Bacteria. Spectrochim. Acta, Part A 2022, 281, 121578 10.1016/j.saa.2022.121578. 35797953

[ref21] Chen Y.-F.; Wang C.-H.; Chang W.-R.; Li J.-W.; Hsu M.-F.; Sun Y.-S.; Liu T.-Y.; Chiu C.-W. Hydrophilic–Hydrophobic Nanohybrids of AuNP-Immobilized Two-Dimensional Nanomica Platelets as Flexible Substrates for High-Efficiency and High-Selectivity Surface-Enhanced Raman Scattering Microbe Detection. ACS Appl. Bio Mater. 2022, 5 (3), 1073–1083. 10.1021/acsabm.1c01151. 35195391

[ref22] Liang S.; Schwartzkopf M.; Roth S. V.; Müller-Buschbaum P. State of the Art of Ultra-thin Gold Layers: Formation Fundamentals and Applications. Nanoscale Adv. 2022, 4, 2533–2560. 10.1039/D2NA00127F. 36132287 PMC9418724

[ref23] Skorikov A.; Albrecht W.; Bladt E.; Xie X.; van der Hoeven J. E. S.; van Blaaderen A.; van Aert S.; Bals S. Quantitative 3D Characterization of Elemental Diffusion Dynamics in Individual Ag@Au Nanoparticles with Different Shapes. ACS Nano 2019, 13, 13421–13429. 10.1021/acsnano.9b06848. 31626527

[ref24] Wang G.; Hao C.; Ma W.; Qu A.; Chen C.; Xu J.; Xu C.; Kuang H.; Xu L. Chiral Plasmonic Triangular Nanorings with SERS Activity for Ultrasensitive Detection of Amyloid Proteins in Alzheimer’s Disease. Adv. Mater. 2021, 33, 2102337 10.1002/adma.202102337. 34309088

[ref25] Li L.; Jiang R.; Shan B.; Lu Y.; Zheng C.; Li M. Near-infrared II Plasmonic Porous Cubic Nanoshells for in Vivo Noninvasive SERS Visualization of Sub-millimeter Microtumors. Nat. Commun. 2022, 13, 5249 10.1038/s41467-022-32975-w. 36068273 PMC9448796

[ref26] Wang J.; Coillet A.; Demichel O.; Wang Z.; Rego D.; Bouhelier A.; Grelu P.; Cluzel B. Saturable plasmonic metasurfaces for laser mode locking. Light: Sci. Appl. 2020, 9, 50 10.1038/s41377-020-0291-2. 32257181 PMC7109045

[ref27] Vinnacombe-Willson G. A.; Conti Y.; Stefancu A.; Weiss P. S.; Cortés E.; Scaramella L. Direct Bottom-Up *In Situ* Growth: A Paradigm Shift for Studies in Wet-Chemical Synthesis of Gold Nanoparticles. Chem. Rev. 2023, 123, 8488–8529. 10.1021/acs.chemrev.2c00914. 37279171 PMC10347433

[ref28] Zheng D.; Pisano F.; Collard L.; Balena A.; Pisanello M.; Spagnolo B.; Mach-Batlle R.; Tantussi F.; Carbone L.; De Angelis F.; Valiente M.; de la Prida L. M.; Ciraci C.; De Vittorio M.; Pisanello F. Toward Plasmonic Neural Probes: SERS Detection of Neurotransmitters through Gold-Nanoislands-Decorated Tapered Optical Fibers with Sub-10 nm Gaps. Adv. Mater. 2023, 35, 2200902 10.1002/adma.202200902. 36479741

[ref29] Khitous A.; Molinaro C.; Gree S.; Haupt K.; Soppera O. Plasmon-Induced Photopolymerization of Molecularly Imprinted Polymers for Nanosensor Applications. Adv. Mater. Interfaces 2023, 10, 2201651 10.1002/admi.202201651.

[ref30] Pekdemir S.; Torun I.; Sakir M.; Ruzi M.; Rogers J. A.; Onses M. S. Chemical Funneling of Colloidal Gold Nanoparticles on Printed Arrays of End-Grafted Polymers for Plasmonic Applications. ACS Nano 2020, 14, 8276–8286. 10.1021/acsnano.0c01987. 32569462

[ref31] Atlan C.; Chatelier C.; Martens I.; Dupraz M.; Viola A.; Li N.; Gao L.; Leake S. J.; Schulli T. U.; Eymery J.; Maillard F.; Richard M. I. Imaging the Strain Evolution of a Platinum Nanoparticle Under Electrochemical Control. Nat. Mater. 2023, 22, 754–761. 10.1038/s41563-023-01528-x. 37095227

[ref32] Awasthi V.; Malik P.; Goel R.; Srivastava P.; Dubey S. K. Nanogap-Rich Surface-Enhanced Raman Spectroscopy-Active Substrate Based on Double-Step Deposition and Annealing of the Au Film over the Back Side of Polished Si. ACS Appl. Mater. Interfaces 2023, 15, 10250–10260. 10.1021/acsami.2c21378. 36757206

[ref33] Kozioł R.; Łapiński M.; Syty P.; Sadowski W.; Sienkiewicz J. E.; Nurek B.; Maraloiu V. A.; Kościelska B. Experimental Tuning of AuAg Nanoalloy Plasmon Resonances Assisted by Machine Learning Method. Appl. Surf. Sci. 2021, 567, 150802 10.1016/j.apsusc.2021.150802.

[ref34] Li Q.; Chen F.; Kang J.; Su J.; Huang F.; Wang P.; Yang X.; Hou Y. Physical Unclonable Anticounterfeiting Electrodes Enabled by Spontaneously Formed Plasmonic Core–Shell Nanoparticles for Traceable Electronics. Adv. Funct. Mater. 2021, 31, 2010537 10.1002/adfm.202010537.

[ref35] Yasuhara A.; Sannomiya T. Atomically Localized Ordered Phase and Segregation at Grain Boundaries in Au–Ag–Cu Ternary Alloy Nanoparticles. J. Phys. Chem. C 2022, 126, 1160–1167. 10.1021/acs.jpcc.1c07816.

[ref36] Gentili D.; Giulia F.; Francesco V.; Massimiliano C.; Fabio B. Applications of Dewetting in Micro and Nanotechnology. Chem. Soc. Rev. 2012, 41, 4430–4443. 10.1039/c2cs35040h. 22491348

[ref37] Yang K.; Yao X.; Liu B.; Ren B. Metallic Plasmonic Array Structures: Principles, Fabrications, Properties, and Applications. Adv. Mater. 2021, 33, 2007988 10.1002/adma.202007988. 34048123

[ref38] Müller-Buschbaum P. The Active Layer Morphology of Organic Solar Cells Probed with Grazing Incidence Scattering Techniques. Adv. Mater. 2014, 26, 7692–7709. 10.1002/adma.201304187. 24677365

[ref39] Abelson A.; Qian C.; Salk T.; Luan Z.; Fu K.; Zheng J. G.; Wardini J. L.; Law M. Collective Topo-epitaxy In the Self-assembly of a 3D Quantum Dot Superlattice. Nat. Mater. 2020, 19, 49–55. 10.1038/s41563-019-0485-2. 31611669

[ref40] Guo R.; Han D.; Chen W.; Dai L.; Ji K.; Xiong Q.; Li S.; Reb L. K.; Scheel M. A.; Pratap S.; Li N.; Yin S.; Xiao T.; Liang S.; Oechsle A. L.; Weindl C. L.; Schwartzkopf M.; Ebert H.; Gao P.; Wang K.; Yuan M.; Greenham N. C.; Stranks S. D.; Roth S. V.; Friend R. H.; Müller-Buschbaum P. Degradation Mechanisms of Perovskite Solar Cells Under Vacuum and One Atmosphere of Nitrogen. Nat. Energy 2022, 7, 459 10.1038/s41560-022-01016-7.

[ref41] Gao L.; Quan L. N.; de Arquer F. P. G.; Zhao Y.; Munir R.; Proppe A.; Quintero-Bermudez R.; Zou C.; Yang Z.; Saidaminov M. I.; Voznyy O.; Kinge S.; Lu Z.; Kelley S. O.; Amassian A.; Tang J.; Sargent E. H. Efficient Near-infrared Light-emitting Diodes Based on Quantum Dots In Layered Perovskite. Nat. Photonics 2020, 14, 227–233. 10.1038/s41566-019-0577-1.

[ref42] Schwartzkopf M.; Rothkirch A.; Carstens N.; Chen Q.; Strunskus T.; Löhrer F. C.; Xia S.; Rosemann C.; Bießmann L.; Körstgens V.; Ahuja S.; Pandit P.; Rubeck J.; Frenzke S.; Hinz A.; Polonskyi O.; Müller-Buschbaum P.; Faupel F.; Roth S. V. *In Situ* Monitoring of Scale Effects on Phase Selection and Plasmonic Shifts during the Growth of AgCu Alloy Nanostructures for Anticounterfeiting Applications. ACS Appl. Nano Mater. 2022, 5, 3832–3842. 10.1021/acsanm.1c04473.

[ref43] Oh H.; Pyatenko A.; Lee M. A Hybrid Dewetting Approach to Generate Highly Sensitive Plasmonic Silver Nanoparticles with a Narrow Size Distribution. Appl. Surf. Sci. 2021, 542, 148613 10.1016/j.apsusc.2020.148613.

[ref44] Li N.; Pratap S.; Korstgens V.; Vema S.; Song L.; Liang S.; Davydok A.; Krywka C.; Müller-Buschbaum P. Mapping structure heterogeneities and visualizing moisture degradation of perovskite films with nano-focus WAXS. Nat. Commun. 2022, 13, 6701 10.1038/s41467-022-34426-y. 36335119 PMC9637205

[ref45] Müller-Buschbaum P. Grazing Incidence Small-Angle X-Ray Scattering: An Advanced Scattering Technique for the Investigation of Nanostructured Polymer Films. Anal. Bioanal. Chem. 2003, 376, 3–10. 10.1007/s00216-003-1869-2. 12734612

[ref46] Guan T.; Chen W.; Tang H.; Li D.; Wang X.; Weindl C. L.; Wang Y.; Liang Z.; Liang S.; Xiao T.; Tu S.; Roth S. V.; Jiang L.; Müller-Buschbaum P. Decoding the Self-Assembly Plasmonic Interface Structure in a PbS Colloidal Quantum Dot Solid for a Photodetector. ACS Nano 2023, 17, 23010–23019. 10.1021/acsnano.3c08526. 37948332

[ref47] Löhrer F. C.; Korstgens V.; Semino G.; Schwartzkopf M.; Hinz A.; Polonskyi O.; Strunskus T.; Faupel F.; Roth S. V.; Müller-Buschbaum P. Following *In Situ* the Deposition of Gold Electrodes on Low Band Gap Polymer Films. ACS Appl. Mater. Interfaces 2020, 12, 1132–1141. 10.1021/acsami.9b17590. 31829550

[ref48] Mitra S.; Basak M. Diverse Bio-sensing and Therapeutic Applications of Plasmon Enhanced Nanostructures. Mater. Today 2022, 57, 225–261. 10.1016/j.mattod.2022.05.023.

[ref49] Gangareddy J.; Rudra P.; Chirumamilla M.; Ganisetti S.; Kasimuthumaniyan S.; Sahoo S.; Jayanthi K.; Rathod J.; Soma V. R.; Das S.; Gosvami N. N.; Krishnan N. M. A.; Pedersen K.; Mondal S.; Ghosh S.; Allu A. R. Multi-Functional Applications of H-Glass Embedded with Stable Plasmonic Gold Nanoislands. Small 2023, 20, 2303688 10.1002/smll.202303688. 37670541

[ref50] Wei W.; Bai F.; Fan H. Oriented Gold Nanorod Arrays: Self-Assembly and Optoelectronic Applications. Angew. Chem., Int. Ed. 2019, 58, 11956–11966. 10.1002/anie.201902620. 30913343

[ref51] Santoro G.; Yu S.; Schwartzkopf M.; Zhang P.; Vayalil S. K.; Risch J. F. H.; Rübhausen M. A.; Hernández M.; Domingo C.; Roth S. V. Silver Substrates for Surface Enhanced Raman Scattering: Correlation Between Nanostructure and Raman Scattering Enhancement. Appl. Phys. Lett. 2014, 104, 243107 10.1063/1.4884423.

[ref52] Park S. G.; Xiao X.; Min J.; Mun C.; Jung H. S.; Giannini V.; Weissleder R.; Maier S. A.; Im H.; Kim D. H. Self-Assembly of Nanoparticle-Spiked Pillar Arrays for Plasmonic Biosensing. Adv. Funct. Mater. 2019, 29, 1904257 10.1002/adfm.201904257.

[ref53] Mao S.; Liu J.; Pan Y.; Lee J.; Yao Z.; Pandey P.; Kunwar S.; Zhu Z.; Shen W.; Belfiore L. A.; Tang J. Morphological and Optical Evolution of Metallic Oxide/Au Nanoparticle Hybrid Thin Film: High Absorption and Reflectance by Plasmonic Enhancement. Appl. Surf. Sci. 2019, 495, 143575 10.1016/j.apsusc.2019.143575.

[ref54] Shao M.; Ji C.; Tan J.; Du B.; Zhao X.; Yu J.; Man B.; Xu K.; Zhang C.; Li Z. Ferroelectrically Modulate the Fermi Level of Graphene Oxide to Enhance SERS Response. Opto-Electron. Adv. 2023, 6 (11), 230094 10.29026/oea.2023.230094.

[ref55] Bai S.; Ren X.; Obata K.; Ito Y.; Sugioka K. Label-Free Trace Detection of Bio-Molecules by Liquid-Interface Assisted Surface-Enhanced Raman Scattering Using a Microfluidic Chip. Opto-Electron. Adv. 2022, 5 (10), 210121 10.29026/oea.2022.210121.

[ref56] Pei Z.; Li J.; Ji C.; Tan J.; Shao Z.; Zhao X.; Li Z.; Man B.; Yu J.; Zhang C. Flexible Cascaded Wire-in-Cavity-in-Bowl Structure for High-Performance and Polydirectional Sensing of Contaminants in Microdroplets. J. Phys. Chem. Lett. 2023, 14 (25), 5932–5939. 10.1021/acs.jpclett.3c00988. 37345745

[ref57] Peng G.-Z.; Hardiansyah A.; Lin H.-T.; Lee R.-Y.; Kuo C.-Y.; Pu Y.-C.; Liu T.-Y. Photocatalytic Degradation and Reusable SERS Detection by Ag Nanoparticles Immobilized on g-C3N4/Graphene Oxide Nanosheets. Surf. Coat. Technol. 2022, 435, 128212 10.1016/j.surfcoat.2022.128212.

[ref58] Buffet A.; Rothkirch A.; Döhrmann R.; Körstgens V.; Abul Kashem M. M.; Perlich J.; Herzog G.; Schwartzkopf M.; Gehrke R.; Müller-Buschbaum P.; Roth S. V. P03, the Microfocus and Nanofocus X-ray Scattering (MiNaXS) Beamline of the PETRA III Storage Ring: the Microfocus Endstation. J. Synchrotron Radiat. 2012, 19, 647–653. 10.1107/S0909049512016895. 22713902 PMC3380660

