# Supplementary material for: High-Power Impulse Magnetron Sputter Deposition of Ag on Self-Assembled Au Nanoparticle Arrays at Low-Temperature Dewetting Conditions
Source: ACS Appl Mater Interfaces. 2024 Jul 16;16(30):40286–96. doi: 10.1021/acsami.4c10726 (PMC11299143; doi:10.1021/acsami.4c10726)
Supplement: Supplementary file 2 — am4c10726_si_002.pdf [file am4c10726_si_002.pdf]

Supporting Information

High-Power Impulse Magnetron Sputter Deposition of Ag on Self-Assembled Au Nanoparticle Arrays at Low-Temperature Dewetting Conditions

Tianfu Guan,[1] Suzhe Liang,[1] Yicui Kang,[2] Evangelina Pensa,[2] Dong Li,[3] Wenkai Liang,[3] Zhiqiang Liang,[3] Yusuf Bulut,[1,4] Kristian A. Reck,[5] Tianxiao Xiao,[1] Renjun Guo,[1] Jonas Drewes,[5] Thomas Strunskus,[5] Matthias Schwartzkopf,[4] Franz Faupel,[5] Stephan V. Roth, [4,6] Emiliano Cortés,[2] Lin Jiang,[3,] and Peter Müller-Buschbaum [1,]**

[1]Technical University of Munich, TUM School of Natural Sciences, Department of Physics, Chair for Functional Materials, James-Franck-Str. 1, 85748 Garching, Germany

[2]Nanoinstitute Munich, Faculty of Physics, Ludwig-Maximilians-Universität München, 80539 München, Germany

[3]Jiangsu Key Laboratory for Carbon-Based Functional Materials & Devices, Institute of Functional Nano & Soft Materials (FUNSOM), Soochow University, Suzhou 215123, P. R. China

[4]Deutsches Elektronen-Synchrotron DESY, Notkestr. 85, 22607 Hamburg, Germany

[5]Chair for Multicomponent Materials, Department of Materials Science, Kiel University, Kaiserstr. 2, 24143 Kiel, Germany

[6]KTH Royal Institute of Technology, Department of Fibre and Polymer Technology, Teknikringen 56-58, SE-100 44 Stockholm, Sweden

Corresponding Author

Lin Jiang: ljiang@suda.edu.cn and Peter Müller-Buschbaum: muellerb@ph.tum.de

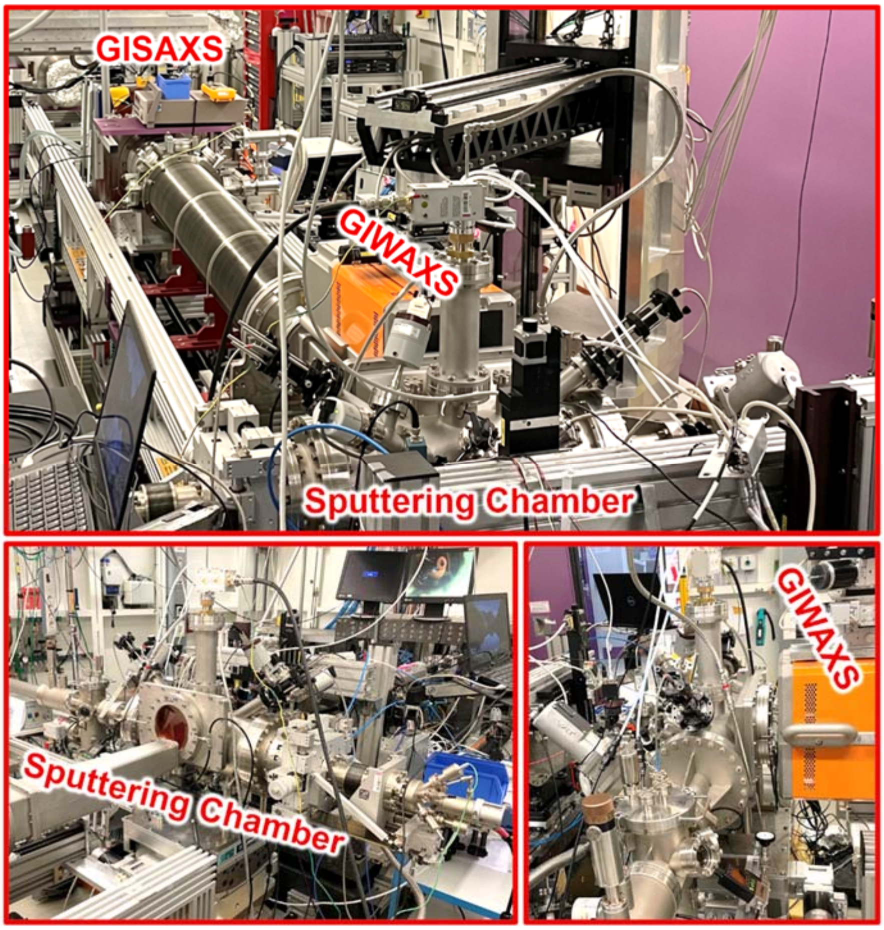

Figure S1. HiPIMS measurement set-up for in situ GISAXS and GIWAXS measurements at the P03 beamline, DESY, Hamburg.

Radius and interparticle distance collection

The radius and interparticle distance were determined from SEM images using the particle analysis module in ImageJ.[1] Figure S2 illustrates the process required to extract particle size and distance distributions from the SEM data (Figure 1b). Initially, the SEM image was cropped to display only well-illuminated, non-overlapping particles. Subsequently, nanoparticles were highlighted to establish the threshold in the software (Figure S2a). For interparticle distribution, 100 random distances from the images were selected, and their distribution is depicted in Figure S2b. A histogram of the particle radius distribution was generated using the diameters of the marked particles (Figure S2c), and the resulting data are presented in Figure 1c.

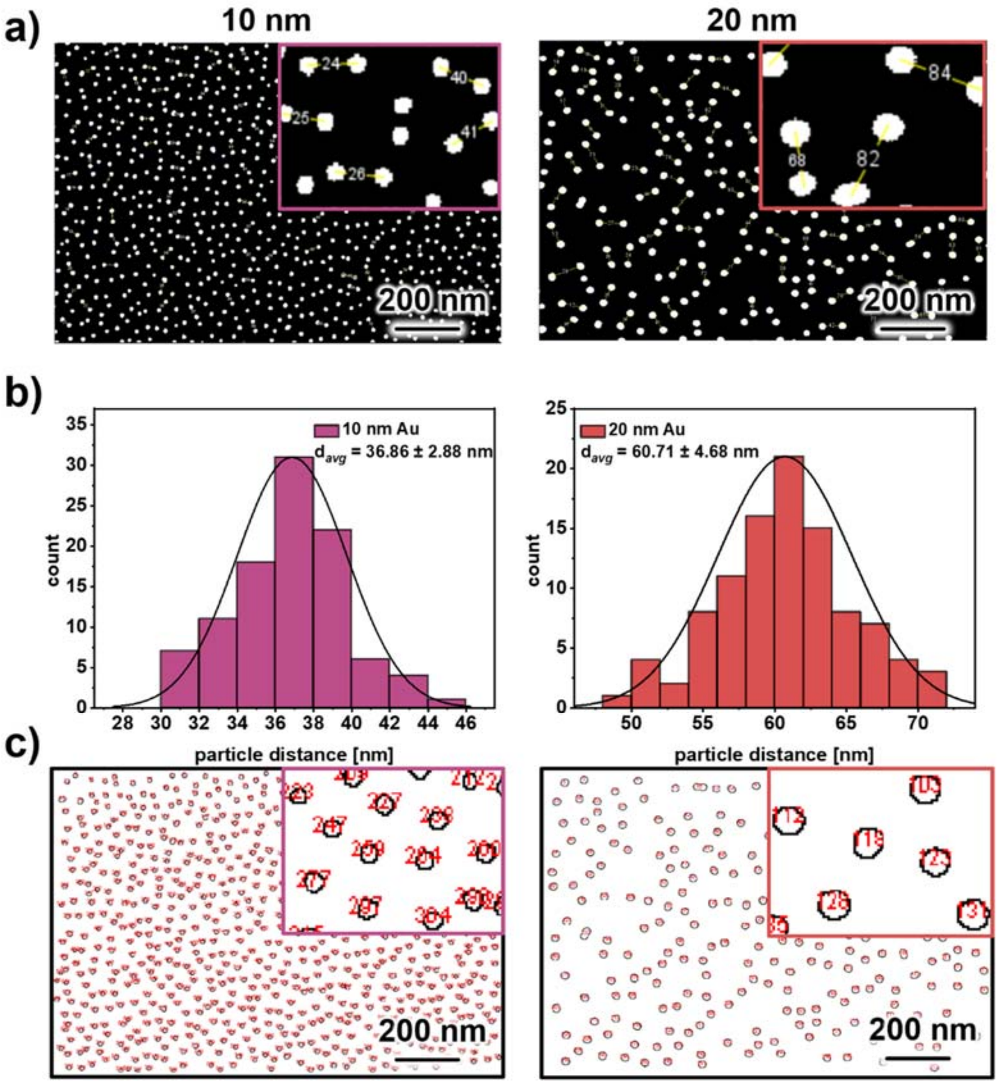

Figure S2. Steps for obtaining the radius and interparticle distance collection from original SEM images (Figure 1b) with ImageJ. a) Thresholded images depicting both 10 nm and 20 nm Au nanoparticles. b) Histogram illustrating the interparticle distribution of 10 nm and 20 nm Au nanoparticles. c) Particles marked of both 10 and 20 nm Au NPs for diameter calculation.

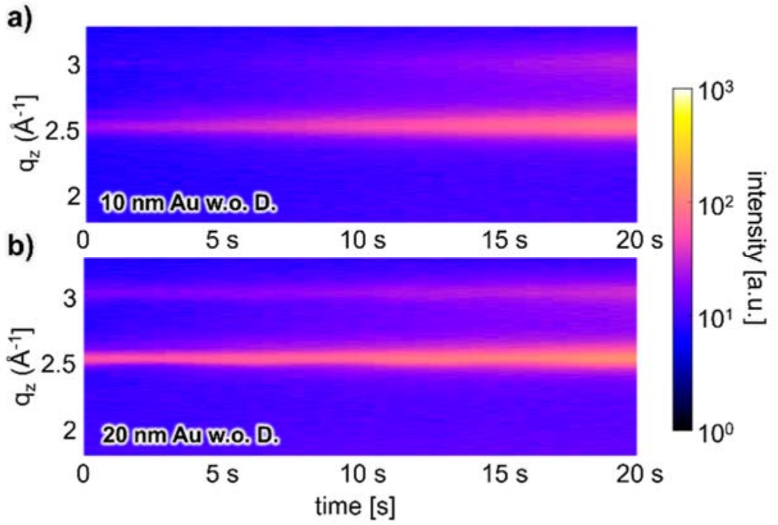

Figure S3. Mapping of vertical line cuts from 2D GIWAXS data measured during the sputter deposition of Ag on (a) 10 nm and (b) 20 nm templates without dewetting conditions.

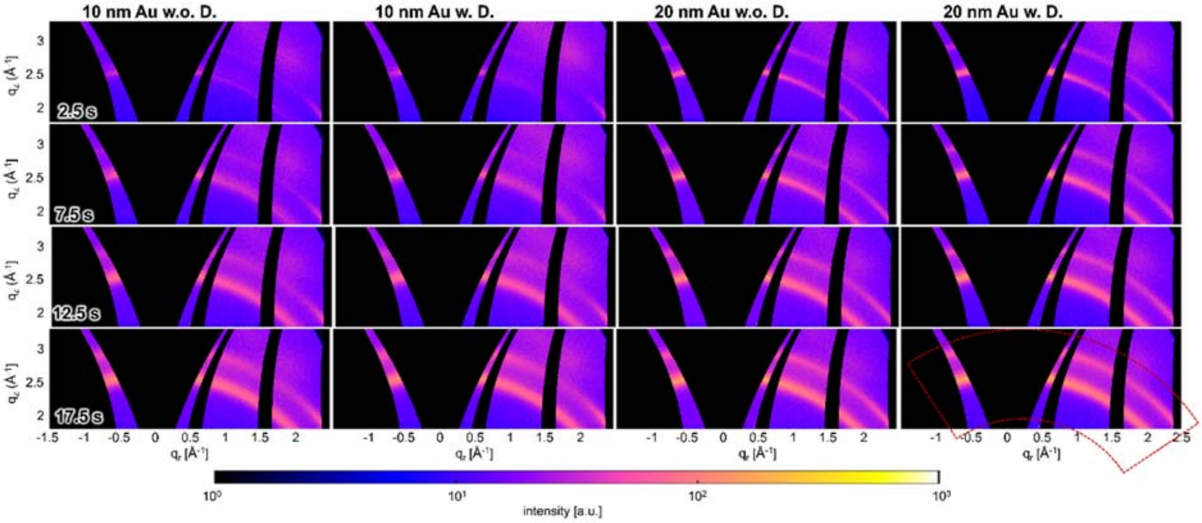

Figure S4. Selected 2D GISAXS data of both 10 and 20 nm Au templates during the sputter deposition of Ag with and without dewetting conditions at selected Ag sputtering times as indicated.

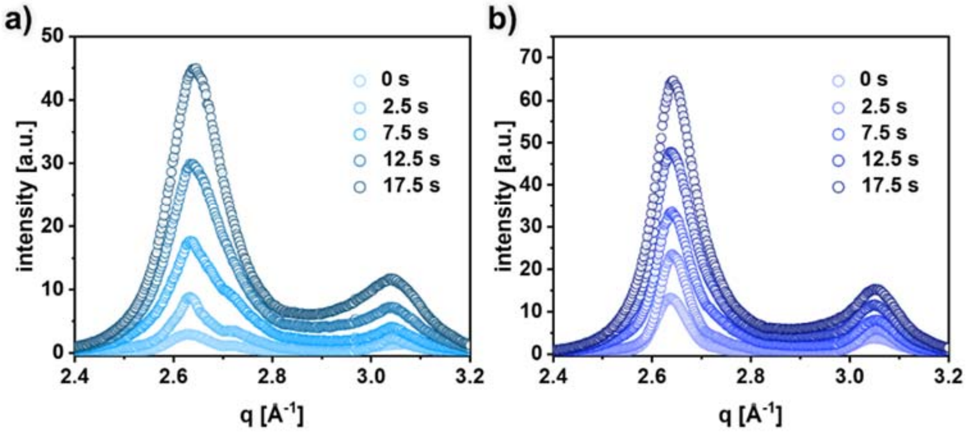

Figure S5. Azimuthal integrations of selected 2D GIWAXS data collected during sputter deposition of Ag on (a) 10 nm and (b) 20 nm templates without dewetting conditions at different sputtering times.

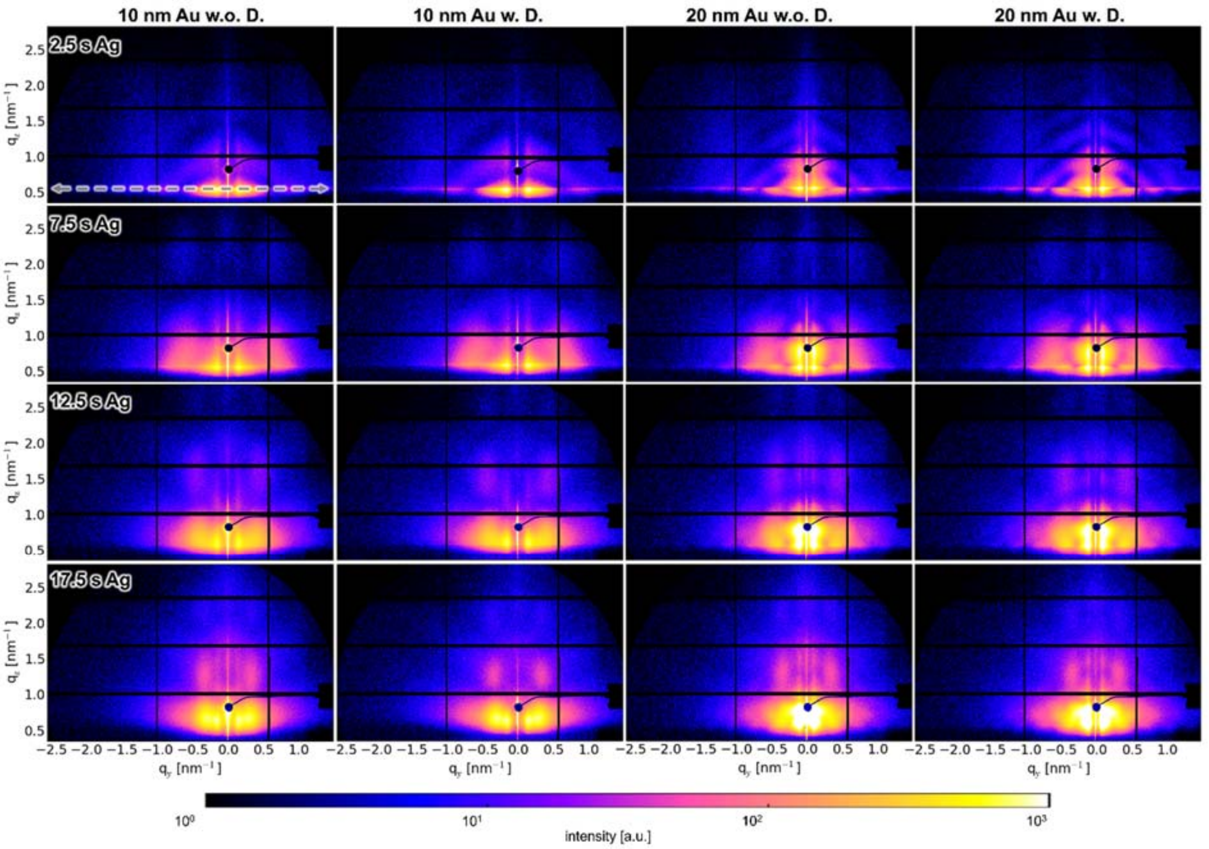

Figure S6. Selected 2D GISAXS data of 10 and 20 nm Au templates during the sputter deposition of Ag with and without dewetting conditions at selected Ag sputtering times as indicated.

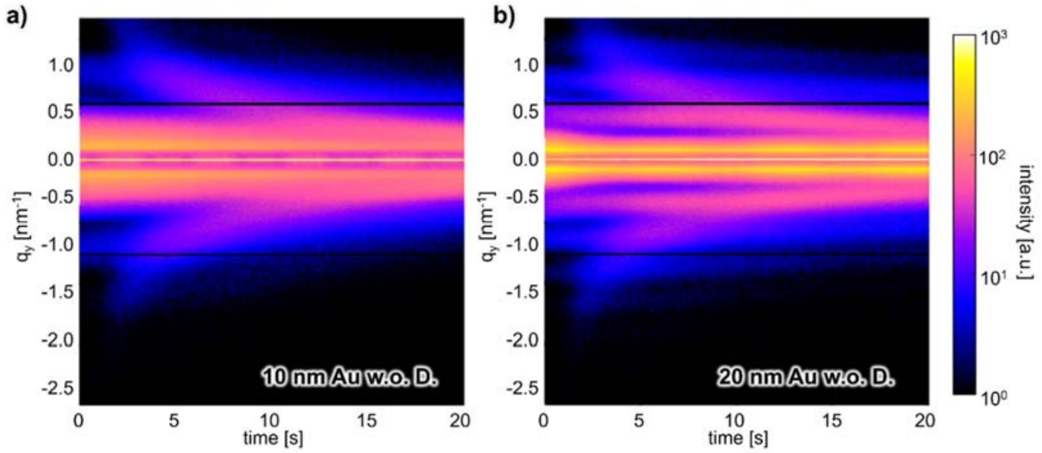

Figure S7. Mapping of horizontal line cuts from 2D GISAXS data of (a) 10 nm and (b) 20 nm templates during the sputter deposition of Ag without dewetting conditions.

GISAXS data modeling of horizontal line cuts

The 2D GISAXS data line cuts are analyzed using a theoretical framework based on the distorted wave Born approximation (DWBA).[2] This approach utilizes the effective interface approximation (EIA) and local monodisperse approximation (LMA) to model the scattering factor, approximating the diffuse scattering using the following equation:

$$P(\vec{q}) \propto \sum_{i} N_i \cdot \langle |F(\vec{q}, R_i)|^2 \rangle \cdot S(\vec{q}, R_i) \qquad \text{(S1)}$$

In the present context, N represents the number of scattering objects, where $F(\vec{q})$ and $S(\vec{q})$ denote the form factor and the structure factor of the scattering objects, respectively. The form factor characterizes the shape of the scattering centers within the structure, which, in the case of the Au/Ag binary system, are attributed to the Au NPs and Ag NPs. The structure factor determined from the fit process accounts for the center-to-center distance between adjacent scattering objects. The overall scattering signal is estimated by incoherently superposing the scattering intensities of individual substructures. Specifically, the proposed model approximates the substructures as standing spheres with a Gaussian size distribution, as illustrated in Figure S6.

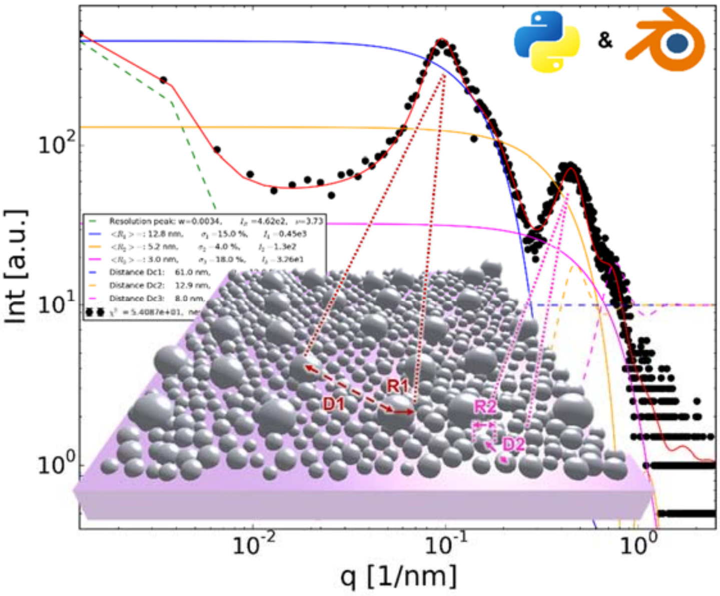

Figure S8. Program interface and fit model of 20 nm Au template/ 10 s Ag under dewetting conditions shown together with data (symbols) and fit (line).

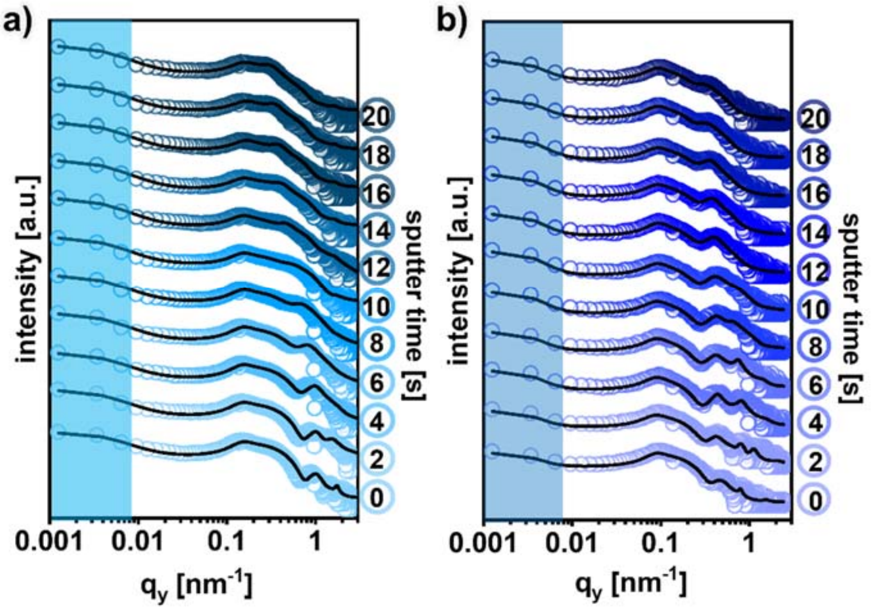

Figure S9. Selected horizontal line cuts from 2D GISAXS data (symbols) are shown together with fits (lines) of both (a) 10 nm and (b) 20 nm templates during the sputter deposition of Ag without dewetting conditions.

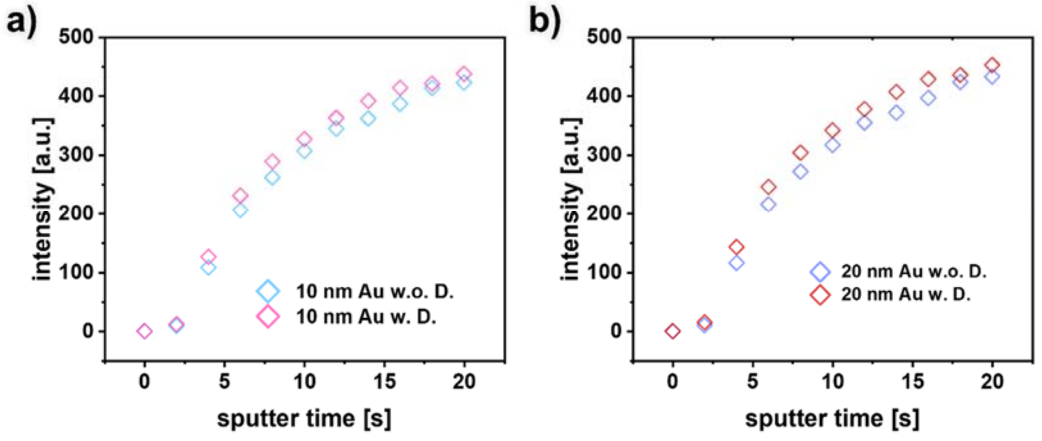

Figure S10. Normalized intensities of the form factors used in the GISAXS modelling of Ag NPs between both (a) 10 and (b) 20 nm Au NP templates during the sputter deposition of Ag with and without dewetting conditions.

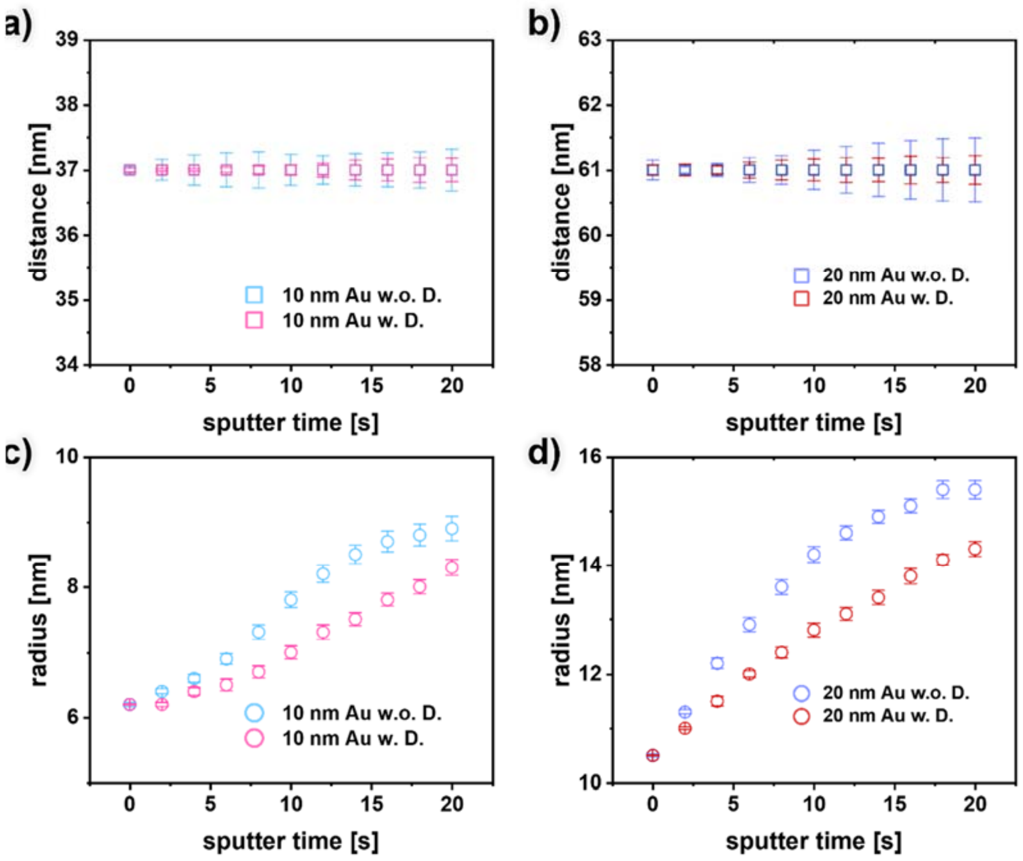

Figure S11. (a, b) Interparticle distance and (c, d) radius of sputtered Au/Ag NPs on both 10 and 20 nm template extracted from GISAXS fit parameters.

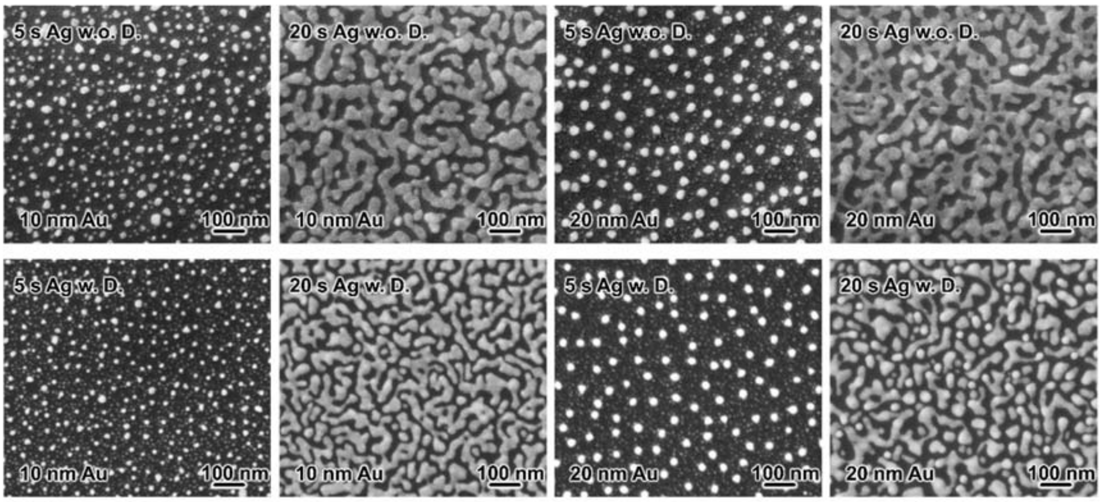

Figure S12. SEM images of 10 nm and 20 nm Au templates with Ag deposited for 5 s and 20 s.

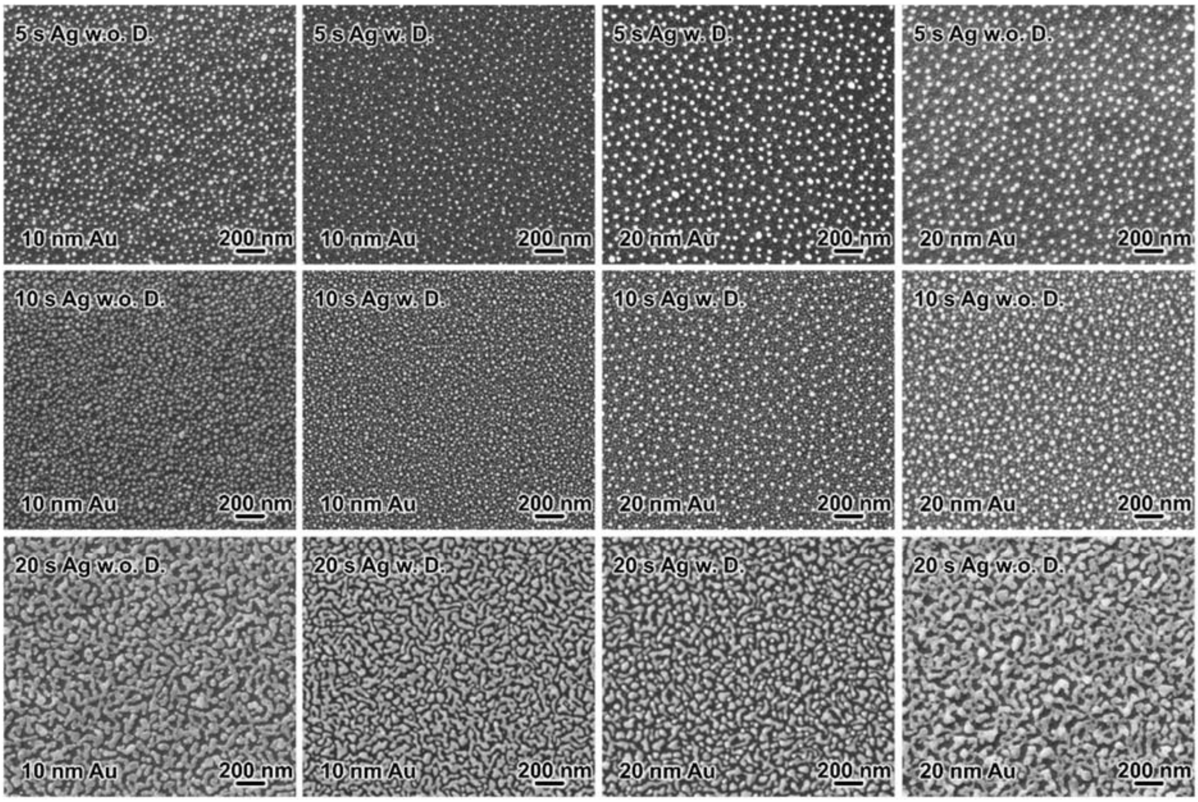

Figure S13. SEM images of both 10 nm and 20 nm Au templates with Ag deposited for 5 s, 10s, and 20 s Ag with and without dewetting conditions.

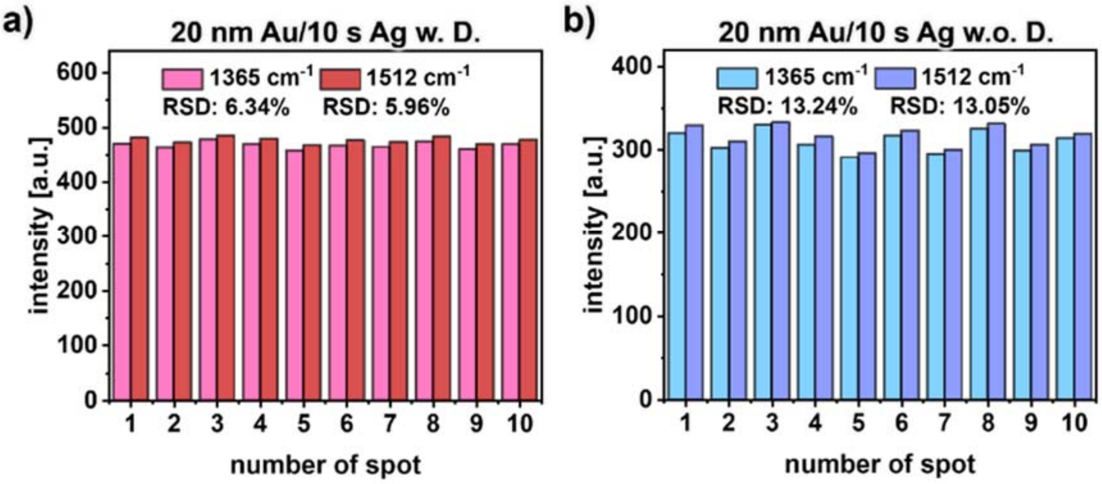

Figure S14. 20 nm Au template deposition Ag for 10s (a) with and (b) without dewetting treatment: RSD of both 1365 cm^{-1} and 1512 cm^{-1} signal of SERS as measured at 10 different spots.

Table S1. Temperature of dewetting treatment from previous work in comparison to our work

Metallic type	Temperature (°C)
Pt[3]	750
Au[4]	500
Au[5]	550
Au/Co[6]	500
Ni/Au[7]	500
Au/Ag[8]	550
Cu/Ag [9]	450
Au/Ag[10]	500
This work	**100**

Table S2. Instrumental parameters of GIXS set up.

	α_i (°)	λ (Å)	SDD (mm)	P (μm)
GISAXS	0.40	1.05	3230	172
GIWAXS	0.40	1.05	193.5	55

References

[1] Schneider, C., Rasband, W. Eliceiri, K. NIH Image to ImageJ: 25 Years of Image Analysis. *Nat. Methods* **2012**, *9*, 671-675.

[2] A. L. Oechsle, J. E. Heger, N. Li, S. Yin, S. Bernstorff, P. Müller-Buschbaum, *ACS Appl. Mater. Interfaces*, **2022**, *14*, 30802.

[3] C. Atlan, C. Chatelier, I. Martens, M. Dupraz, A. Viola, N. Li, L. Gao, S. J. Leake, T. U. Schulli, J. Eymery, F. Maillard, M. I. Richard, *Nat. Mater.* **2023**, *22*, 754.

[4] D. Zheng, F. Pisano, L. Collard, A. Balena, M. Pisanello, B. Spagnolo, R. Mach-Batlle, F. Tantussi, L. Carbone, F. De Angelis, M. Valiente, L. M. de la Prida, C. Ciraci, M. De Vittorio, F. Pisanello, *Adv. Mater.* **2023**, *35*, e2200902.

[5] J. Gangareddy, P. Rudra, M. Chirumamilla, S. Ganisetti, S. Kasimuthumaniyan, S. Sahoo, K. Jayanthi, J. Rathod, V. R. Soma, S. Das, N. N. Gosvami, N. M. A. Krishnan, K. Pedersen, S. Mondal, S. Ghosh, A. R. Allu, *Small* **2023**, *20*, e2303688.

[6] D. A. Garfinkel, N. Tang, G. Pakeltis, R. Emery, I. N. Ivanov, D. A. Gilbert, P. D. Rack, *ACS Appl. Mater. Interfaces* **2022**, *14*, 15047.

[7] Q. Li, F. Chen, J. Kang, J. Su, F. Huang, P. Wang, X. Yang, Y. Hou, *Adv. Funct. Mater.* **2021**, *31*, 2010537.

[8] R. Kozioł, M. Łapiński, P. Syty, W. Sadowski, J. E. Sienkiewicz, B. Nurek, V. Adrian Maraloiu, B. Kościelska, *Appl. Surf. Sci.* **2021**, *567*, 15082.

[9] M. Bubaš, V. Janicki, S. A. Mezzasalma, M. C. Spadaro, J. Arbiol, J. Sancho-Parramon, *Appl. Surf. Sci.* **2021**, *564*,150260.

[10] S. Kunwar, S. Pandit, J. H. Jeong, J. Lee, *Nano Micro-Lett.* **2020**, *12*, 91.
